# 20E-mediated regulation of *BmKr-h1* by BmKRP promotes oocyte maturation

**DOI:** 10.1186/s12915-021-00952-2

**Published:** 2021-02-25

**Authors:** Zidan Zhu, Chunmei Tong, Binbin Qiu, Hongguang Yang, Jiahui Xu, Sichun Zheng, Qisheng Song, Qili Feng, Huimin Deng

**Affiliations:** 1 Guangdong Key Laboratory of Insect Developmental Biology and Applied Technology, Guangzhou Key Laboratory of Insect Development Regulation and Application Research, Institute of Insect Science and Technology & School of Life Sciences, South China Normal University, Guangzhou, 510631 China; 2 Division of Plant Sciences, University of Missouri, Columbia, MO 65211 USA

**Keywords:** 20-hydroxyecdysone, *Krüppel homolog 1*, *Kr-h1* regulatory protein, Vitellogenin receptor, Oogenesis

## Abstract

**Background:**

Krüppel homolog 1 (Kr-h1) is a critical transcription factor for juvenile hormone (JH) signaling, known to play a key role in regulating metamorphosis and adult reproduction in insects. Kr-h1 can also be induced by molting hormone 20-hydroxyecdysone (20E), however, the underlying mechanism of 20E-induced *Kr-h1* expression remains unclear. In the present study, we investigated the molecular mechanism of *Kr-h1* induction by 20E in the reproductive system of a model lepidopteran insect, *Bombyx mori*.

**Results:**

Developmental and tissue-specific expression analysis revealed that *BmKr-h1* was highly expressed in ovaries during the late pupal and adult stages and the expression was induced by 20E. RNA interference (RNAi)-mediated depletion of *BmKr-h1* in female pupae severely repressed the transcription of *vitellogenin receptor* (*VgR*), resulting in the reduction in vitellogenin (Vg) deposition in oocytes. BmKr-h1 specifically bound the Kr-h1 binding site (KBS) between − 2818 and − 2805 nt upstream of *BmVgR* and enhanced the transcription of *BmVgR*. A 20E *cis*-regulatory element (CRE) was identified in the promoter of *BmKr-h1* and functionally verified using luciferase reporter assay, EMSA and DNA-ChIP. Using pull-down assays, we identified a novel transcription factor *B. mori Kr-h1* regulatory protein (BmKRP) that specifically bound the *BmKr-h1* CRE and activated its transcription. CRISPR/Cas9-mediated knockout of *BmKRP* in female pupae suppressed the transcription of *BmKr-h1* and *BmVgR*, resulting in arrested oogenesis.

**Conclusion:**

We identified BmKRP as a new transcription factor mediating 20E regulation of *B. mori* oogenesis. Our data suggests that induction of *BmKRP* by 20E regulates *BmKr-h1* expression, which in turn induces *BmVgR* expression to facilitate Vg uptake and oogenesis.

**Supplementary Information:**

The online version contains supplementary material available at 10.1186/s12915-021-00952-2.

## Background

Juvenile hormone (JH) and 20-hydroxyecdysone (20E) regulate many physiological processes, including the development, metamorphosis and reproduction of insects [1]. During larval stages, JH binds its receptor Methoprene-tolerant (MET) to initiate the JH signaling pathway, suppressing metamorphosis until larvae reach the appropriate size and development stage. In the final instar, JH titers decrease while 20E levels increase to induce larval-pupal metamorphosis [1–3]. JH and 20E also regulate many aspects of reproduction, including previtellogenic development, vitellogenesis and oogenesis [4–7]. Vitellogenesis is a critical event of female reproduction, in which vitellogenin (Vg) is synthesized in fat body, secreted into hemolymph and then taken up by developing oocytes via receptor-mediated endocytosis [8, 9].

Krüppel homolog 1 (Kr-h1), a transcription factor containing a DNA binding domain with eight cystine 2 histidine 2 (C_2_H_2_)-type zinc (Zn)-fingers, is induced by JH and functions in the JH signal transduction pathway. The properties of *Kr-h1* as a transducer of the anti-metamorphic action of JH are conserved in hemimetabolous and holometabolous insects [10–15]. *Kr-h1* can also be induced by 20E, and in fact, *Kr-h1* was first identified as a stage-specific modulator of the prepupal ecdysone response at the onset of *Drosophila melanogaster* metamorphosis [16]. *DmKr-h1* was upregulated 2-fold at 6 h post 20E treatment and 3.5-fold at 6 h post 20E and cycloheximide treatment in the cultured partial blue gut of 3rd instar larvae [17]. Exposure of *Tribolium castaneum* cells to 10 μM 20E induced the expression of *Kr-h1* [18]. In *Helicoverpa armigera*, *HaKr-h1* was upregulated 2.0-fold at 24 h post 20E treatment in the 4th instar larval epidermis [19]. Treatment of 20E in the early 4th larval instar of the recessive trimolter European No. 7 mutant of *Bombyx mori* maintains continuous expression of *BmKr-h1*, suppresses premature induction of broad-complex (*BR-C Z1*), and then induced an additional larval instar [20]. In *B. mori*, 20E synergized *BmKr-h1* induction by JH analog (JHA) methoprene in the cultured epidermis of 4th instar larvae and pupae [3, 15]. Based on the above data, the induced expression of *Kr-h1* by 20E exists in a variety of insects. However, the molecular mechanisms and physiological functions of 20E-mediated *BmKr-h1* induction remain unknown.

In addition to repressing metamorphosis, *Kr-h1* has also been found to function in female reproduction. In *Locusta migratoria*, *Nilaparvata lugens*, *Aedes aegypti*, *H. armigera*, *Bactrocera dorsalis*, and *Sogatella furcifera*, silencing *Kr-h1* blocks JH-regulated expression of Vg, oocyte maturation and ovarian development, and reduces egg production [19, 21–25]. However, in *Pyrrhocoris apterus* and *T. castaneum*, although depletion of *Met* suppresses the *Vg* expression in fat body, silencing *Kr-h1* has no effect on *Vg* expression [14, 26]. In *Cimex lectularius*, knocking down of *Kr-h1* in adult females does not reduce the number of eggs but severely affects egg hatching [27]. Thus, the function of *Kr-h1* in female reproduction varies widely among insect species, and the molecular mechanism of *Kr-h1* in reproduction remains largely unknown.

In the present study, a 20E *cis*-regulatory element (CRE) in the promoter region of *BmKr-h1* and a novel *Kr-h1* regulatory protein (BmKRP) that binds this CRE were identified. Furthermore, in responding to 20E stimulation, BmKRP was shown to induce *BmKr-h1* expression, which in turn regulated ovarian development and oogenesis via Vg receptor (BmVgR)*.* Our data demonstrated that 20E-induced BmKRP mediated *Kr-h1* expression in the female reproduction of *B. mori*.

## Results

### Developmental and 20E-induced expression of *BmKr-h1* in ovaries

**Fig. 1:**
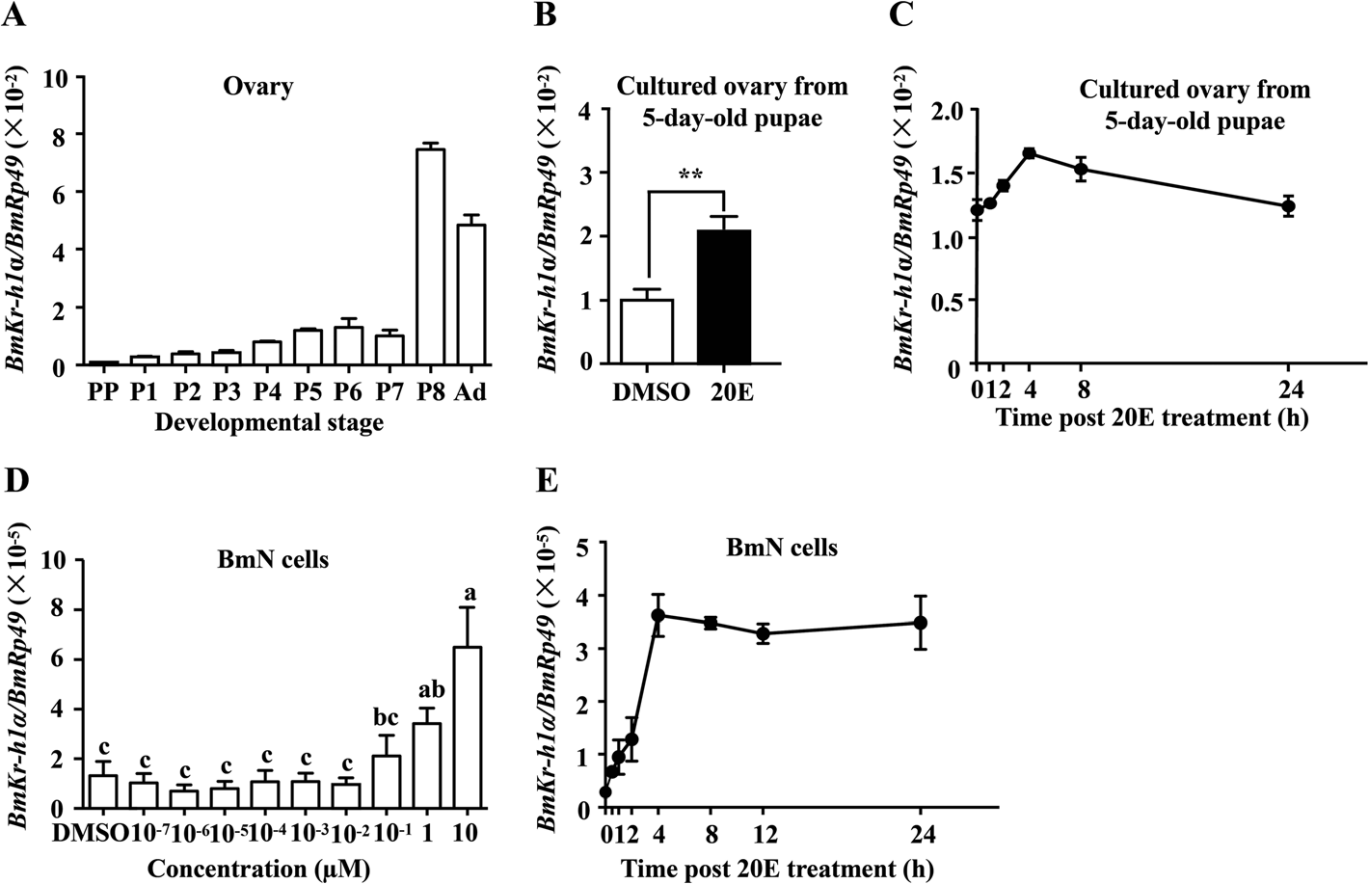
Developmental and 20E-induced expression of *BmKr-h1* in cultured ovaries and BmN cells. **a** Developmental expression profiles of *BmKr-h1* in the ovaries. **b**, **c** Induced expression and temporal changes in *BmKr-h1* at 2 h post 20E treatment (1 μM) in the cultured ovaries from 5-day-old pupae. **d**, **e** Dose-dependent and time-dependent expression of *BmKr-h1* expression in BmN cells. Data shown is mean ± SD (*n* = 3) and the individual data values are shown in Additional file 2. Different letters above the columns indicate significant differences at *p* < 0.05 by ANOVA. The significance of the differences between the treatment and control was statistically analyzed at *p* < 0.05 (*), *p* < 0.01 (**), and *p* < 0.001 (***) using *t* test. P, pupal stage; PP, prepupal stage; Pn, day n of pupal stage

The developmental and 20E-induced expression profiles of *BmKr-h1* in the ovaries were determined by quantitative real-time PCR (qRT-PCR). Two isoforms of *BmKr-h1*, *BmKr-h1α* (AB360766) and *BmKr-h1β* (AB642242), have been reported [28]. Compared to *BmKr-h1β*, *BmKr-h1α* is predominant in the larval epidermis and is induced by JHA in the epidermis of day 0 of 4th instar allatectomized larvae and in NIAS-Bm-aff3 cells, but *BmKr-h1β* is not sensitive to JHA induction [28]. The expression of *BmKr-h1α* was also detected predominantly in the ovaries comparing to *BmKr-h1β* (Additional file 1, Fig. S1A and B) and its mRNA levels were low in the prepupal stage, gradually increased until day 7 of the pupal stage, then dramatically increased on day 8 of pupal stage and remained high during the adult stage (Fig. 1a), which are in general correlated with the peaks of 20E titers as reported previously [29, 30]. Since JH is absent during the late pupal stage, these results suggest that *BmKr-h1α* might be induced by 20E through an unknown transcription factor. To test this hypothesis, the effect of 20E on the expression of *BmKr-h1* was examined in the cultured ovaries isolated from 5-day-old pupae and BmN cells. The result showed clearly that the expression of *BmKr-h1α* was induced by 20E at a physiological concentration of 1 μM in the cultured ovaries and reached the peak at 4 h post 20E treatment (Fig. 1b, c). In BmN cells, 20E induced *BmKr-h1α* in a time- and dose-dependent manner (Fig. 1d, e). Although the *BmKr-h1β* expression was also induced by 20E treatment in the cultured ovaries and BmN cells, the mRNA levels were much lower than those of *BmKr-h1α* (Additional file 1, Fig. S1B and C). Therefore, *BmKr-h1α* was used for in-depth studies in the subsequent experiments.

### *BmKr-h1* upregulated the *BmVgR* transcription in the oocyte development

**Fig. 2:**
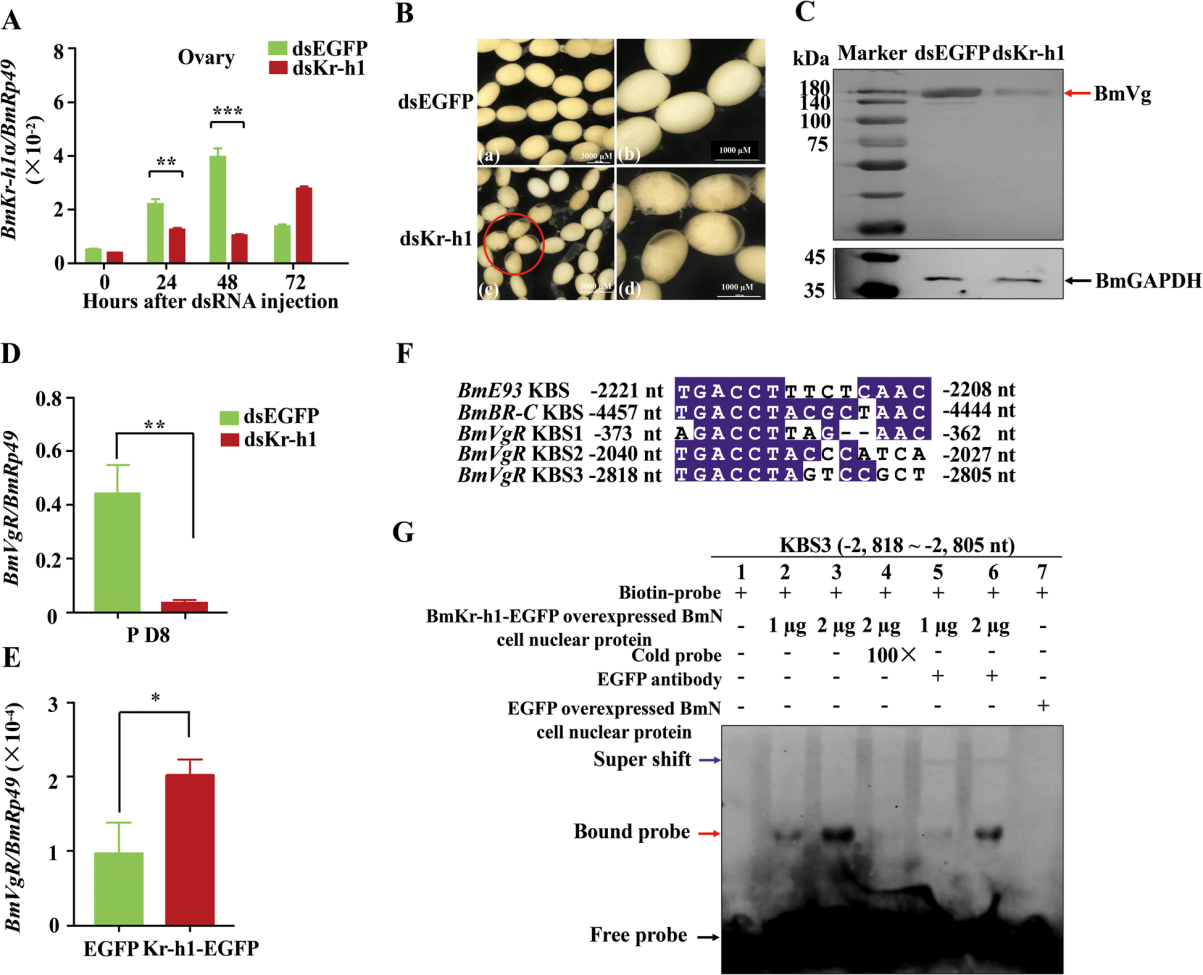
Effects of RNAi mediated silencing of *BmKr-h1* on the *BmVgR* expression and the oocyte development. A total of 10 μg of dsRNA per pupae was injected into the intersegmental region in the abdomen of 6-day-old pupae. **A** qRT-PCR analysis of *BmKr-h1* mRNA in the ovaries of the treated pupae. **B** Phenotypes of oocytes in the ovaries of the dsRNA-treated pupae at day 8. **C** Western blot analysis of BmVg protein in the oocyte at 48 h post dsRNA treatment. A total of 15 μg protein was loaded per lane and probed with anti-BmVg and anti-GAPDH antibodies, respectively. **D** qRT-PCR analysis of the expression change of *BmVgR* in the oocyte. **E** qRT-PCR detection of the effect of *BmKr-h1* overexpression on the transcript level of *BmVgR*. **F** Prediction of KBS in the *BmVgR* promoter based on the KBS core sequences of the *BmBR-C* and *BmE93* promoters [3, 31]. The conserved amino acid residues are in blue background. **G** EMSA of the binding of the nuclear protein from BmN cells overexpressing BmKr-h1-EGFP with the KBS3 in the *BmVgR* promoter. For qRT-PCR, *Rp49* amplified was used as the internal control. Significance of the differences between the treatment and control was statistically analyzed at *p* < 0.05 (*), *p* < 0.01 (**), and *p* < 0.001 (***) using *t* test. Dn, day of development; P, pupal stage

To investigate the function of *BmKr-h1* induced by 20E in the female reproduction, *BmKr-h1* dsRNA was injected into the wild-type pupae on day 6. qRT-PCR analysis showed that the *BmKr-h1* dsRNA efficiently reduced *BmKr-h1* transcript abundance in the ovaries 24 and 48 h post dsRNA treatment (Fig. 2A). Totally, 13% of the oocytes in the ovaries of ds*BmKr-h1*-treated 8-day-old pupae had less yolk protein deposition and partially transparent chorion, whereas there were none of these abnormal oocytes in the ovaries of the control (dsEGFP treated) (Fig. 2B and Additional file 1, Table S1). These abnormal eggs gradually became black and the embryos inside the eggs died after oviposition (Additional file 1, Fig. S2), whereas the oocytes in the eggs injected with dsGFP were filled with normally developing embryos (Additional file 1, Fig. S2), results similar to that in our previous report [32]. Western blotting analysis showed that Vg levels were reduced in the ovaries of pupae on day 8 post ds*BmKr-h1* treatment, compared to the control (Fig. 2C). Approximately 78% of the ds*BmKr-h1*-treated adults laid eggs that eventually turned black, whereas only 15% of the dsGFP-treated adults did so. The hatch rate of the eggs laid by the ds*BmKr-h1*-treated adults was reduced by approximately 7% (Additional file, Table S1). However, the numbers of eggs laid by the females showed no obvious difference between the dsBmKr-h1-treated and dsGFP-treated adults (Additional file 1, Table S1). These results suggest that the suppression of *BmKr-h1* in late pupae inhibits the deposition of Vg in the ovary, thus preventing oocyte maturation.

To further determine how BmKr-h1 regulates Vg deposition, the expression of the Vg receptor *BmVgR*, which is responsible for the uptake of Vg into the oocytes, was examined in the oocytes of the ds*BmKr-h1*-treated pupae. qRT-PCR analysis showed that silencing of *BmKr-h1* dramatically decreased the *BmVgR* expression in the oocytes of ds*BmKr-h1*-treated pupae compared to the controls (Fig. 2D). Furthermore, the levels of *BmVgR* and BmVg were low in the ovaries during the early pupal stage, gradually increased during the mid-pupal stage, and then rapidly increased during the late pupal and adult stages (Additional file 1, Fig. S3A and E), which was coincident with the expression patterns of *BmKr-h1* in the ovaries (Fig. 1a). The *BmVgR* expression in the cultured ovaries from 5-day-old pupae and BmN cells was also upregulated by 20E in a dose- and time-dependent manner (Additional file 1, Fig. S3B and Fig. S3C-D). Furthermore, the *BmVgR* expression was increased when *BmKr-h1* was overexpressed in BmN cells (Fig. 2E).

To determine whether the expression of *BmVgR* was regulated directly by BmKr-h1, the promoter of *BmVgR* was searched for the presence of a potential Kr-h1 binding site (KBS) sequence similar to the KBS homologous sequences in the promoters of the transcription factors *E93* and *BR-C* [3, 31]. Three potential KBS sites (− 373 ~ − 362 nt, − 2040 ~ − 2027 nt, and − 2818 ~ − 2805 nt) similar to the conserved motif GACCT were detected in the upstream region of the transcriptional start site of *BmVgR* (Fig. 2F). Electrophoretic mobility shift assay (EMSA) analysis showed that the labeled − 2818 ~ − 2805 nt probe containing KBS specifically bound the nuclear proteins from BmKr-h1-EGFP overexpressing cells (Fig. 2G, lane 2–3), and 100-fold cold probe eliminated the binding (Fig. 2G, lane 4), whereas the labeled − 373 ~ − 362 nt and − 2040 ~ − 2027 nt probes did not bind nuclear proteins from BmKr-h1-EGFP overexpressing cells (Additional file, Fig. S4). When the BmKr-h1-EGFP-overexpressing nuclear proteins were incubated with the anti-EGFP polyclonal antibody, a weak supershifted binding signal was detected (Fig. 2G, lane 5–6), suggesting that the protein bound the KBS is BmKr-h1. These results suggest that BmKr-h1 directly binds the KBS (− 2818 ~ − 2805 nt) in the promoter region of *BmVgR* and upregulates its transcription, in turn enhancing Vg uptake by oocytes.

### Identification of 20E CREs in the *BmKr-h1* promoter

**Fig. 3:**
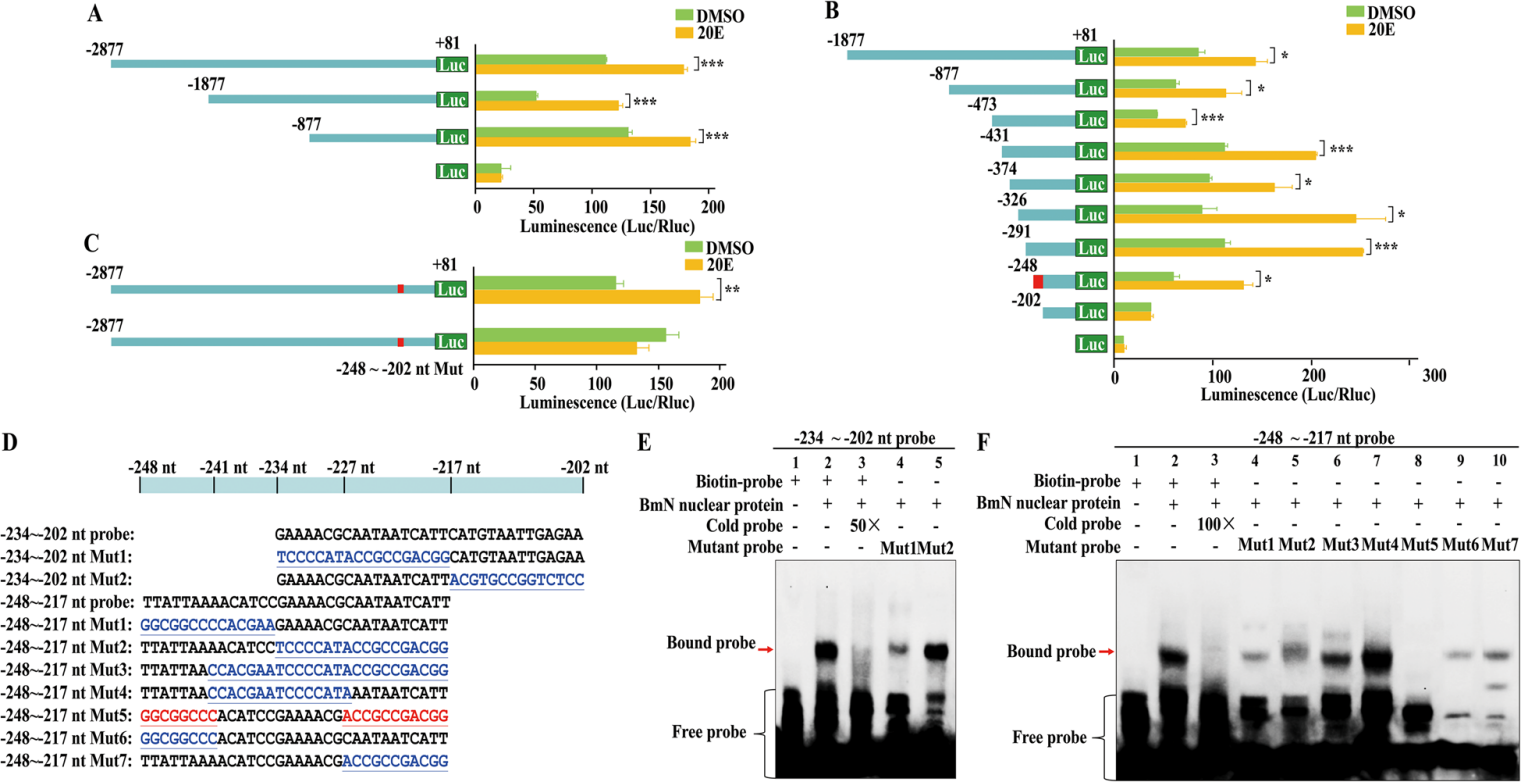
Identification of 20E CRE in *BmKr-h1* promoter. **a**, **b** Reporter plasmids containing the 5′-flanking and first intron regions of *BmKr-h1* were assayed. **c** Effect of mutation of the putative 20E CRE on the *BmKr-h1* promoter activity. The region of the putative 20E CRE is indicated by a red box. **d** The sequence and the mutated sequences of the region between − 248 ~ − 202 nt of the *BmKr-h1* promoter. The underlined nucleotides in blue and red represent the mutated nucleotides. **e** EMSA analysis of binding of nuclear proteins extracted from BmN cells using a region between − 234 ~ − 202 nt and the mutated probes. **f** EMSA of the binding of nuclear proteins extracted from BmN cells with the probe of the region between − 248 ~ − 217 nt and the mutated probes. The data shown is mean ± SD (*n* = 3) and the individual data values are shown in Additional file 2. The significance of the differences between the treatment and control was statistically analyzed at *p* < 0.05 (*), *p* < 0.01 (**), and *p* < 0.001 (***) using *t* test

To further explore the responsiveness of *BmKr-h1* to 20E, the 20E *cis*-regulatory region (20 CRE) in the *BmKr-h1* promoter was identified using the dual-luciferase reporter assays. Three regions of *BmKr-h1* promoter (− 2877 ~ + 81 nt, − 1877 ~ + 81 nt, and − 877 ~ + 81 nt) were cloned into pGL3-basic vectors and tested. Compared to pGL3-basic vector, all three constructs showed a significant activation of the luciferase reporter in the presence of 20E, suggesting that the region between − 877 ~ + 81 nt is necessary for the 20E response (Fig. 3a). Thus, further truncated constructs within the − 877 ~ + 81 nt region (− 473 ~ + 81 nt, − 431 ~ + 81 nt, − 374 ~ + 81 nt, − 326 ~ + 81 nt, − 291 ~ + 81 nt, − 248 ~ + 81 nt, and − 202 ~ + 81 nt) were prepared and tested. The luciferase activity assays showed that the promoter activity of all these fragments was activated by 20E, except the shortest region, − 202 ~ + 81 nt (Fig. 3b), implying that the region between − 248 and − 202 nt is crucial for 20E response. To further confirm whether the − 248 ~ − 202 nt sequence of the *BmKr-h1* promoter is responsible for 20E induction, mutations were introduced in this region (TTATTAAAACATCCGAAAACGCAATAATCATTCATGTAATTGAGAA were mutated to different nucleotides GGCGGCCCCACGAATCCCCATACCGCCGACGGACGTGCCGGTCTCC) and evaluated. The wild-type construct showed an activation in luciferase activity in the presence of 20E, whereas the construct containing the mutant sequence of the region between − 248 and − 202 nt showed no response to 20E treatment (Fig. 3c). Thus, the region between − 248 and − 202 nt of the *BmKr-h1* promoter is demonstrated to be critical for 20E response.

To accurately pinpoint the nucleotide sequences within the − 248 ~ − 202 nt region required for response to 20E, EMSA was performed using biotin end-labeled double-stranded probes containing the − 248 ~ − 202 nt region and nuclear proteins extracted from 20E-treated BmN cells. Eleven probes, three for the − 234 ~ − 202 nt region and eight for the − 248 ~ − 217 nt region along with different mutations were prepared (Fig. 3d). BmN cell nuclear proteins were able to bind the wild-type probes and the binding was competed off by 50-fold (Fig. 3e, lane 3) and 100-fold (Fig. 3f, lane 3) unlabeled probes. The binding signal was weakened when the mutant probe 1 (TCCCCATACCGCCGACGGCATGTAATTGAGAA, the nucleotides between − 234 and − 217 nt were mutated in the 234 ~ − 202 nt region) was used. However, the binding was still detected for mutant probe 2 (GAAAACGCAATAATCATTACGTGCCGGTCTCC, the nucleotides between − 217 and − 202 nt were mutated in the − 234 to − 202 nt region (Fig. 3e, lane 4–5), suggesting that the region (GAAAACGCAATAATCATT) is essential for binding, whereas the region (CATGTAATTGAGAA) is not. In addition, the binding signal was weakened when the mutant probes 1, 2, and 3 of the − 248 ~ − 217 nt region were used (Fig. 3f, lane 4–6), whereas the mutant probe 4 did not alter the binding signal (Fig. 3e, lane 7), suggesting that the region (AACATCCGAAAACGC) is not critical for the binding. We then mutated the flanking sequences of the probe − 248 ~ − 217 nt region individually or simultaneously (Fig. 3d). When the left and right flanking sequences of the − 248 ~ − 217 nt region were mutated simultaneously as shown in the mutant probe 5, the binding signal disappeared (Fig. 3f, lane 8). However, when only the left sequence (the mutant probe 6) or right sequence (the mutant probe 7) was mutated individually, the binding signal was weakened but not completely abolished (Fig. 3f, lane 9–10). These results together suggest that the essential protein-binding sites (or 20E CRE) of the *BmKr-h1* gene are located at − 248 ~ − 241 nt (TTATTAA) and − 227 ~ − 217 nt (AATAATCATT) in the promoter.

We then screened public genomic databases for the sequences homologous to the potential 20E CRE in other insect species. Sequences with similarity to the 20E CRE (TTATTAA or AATAATCATT) were found in the 3-kb upstream regions of *Kr-h1* in *T. castaneum*, *Apis mellifera*, *A. aegypti*, *Acyrthosiphon pisum*, and *D. melanogaster*. All these species possess identical TTATTAA sequences (Additional file 1, Fig. S5A) and AATAATCATT sequences (Additional file 1, Fig. S5B) in their upstream regions. Moreover, like *BmKr-h1*, the AATAATCATT sequence was found adjacent to or partially overlapping with the TTATTAA sequence in their *Kr-h1* promoters in all these species (Additional file 1, Fig. S5).

### Identification of 20E CRE binding protein

**Fig. 4:**
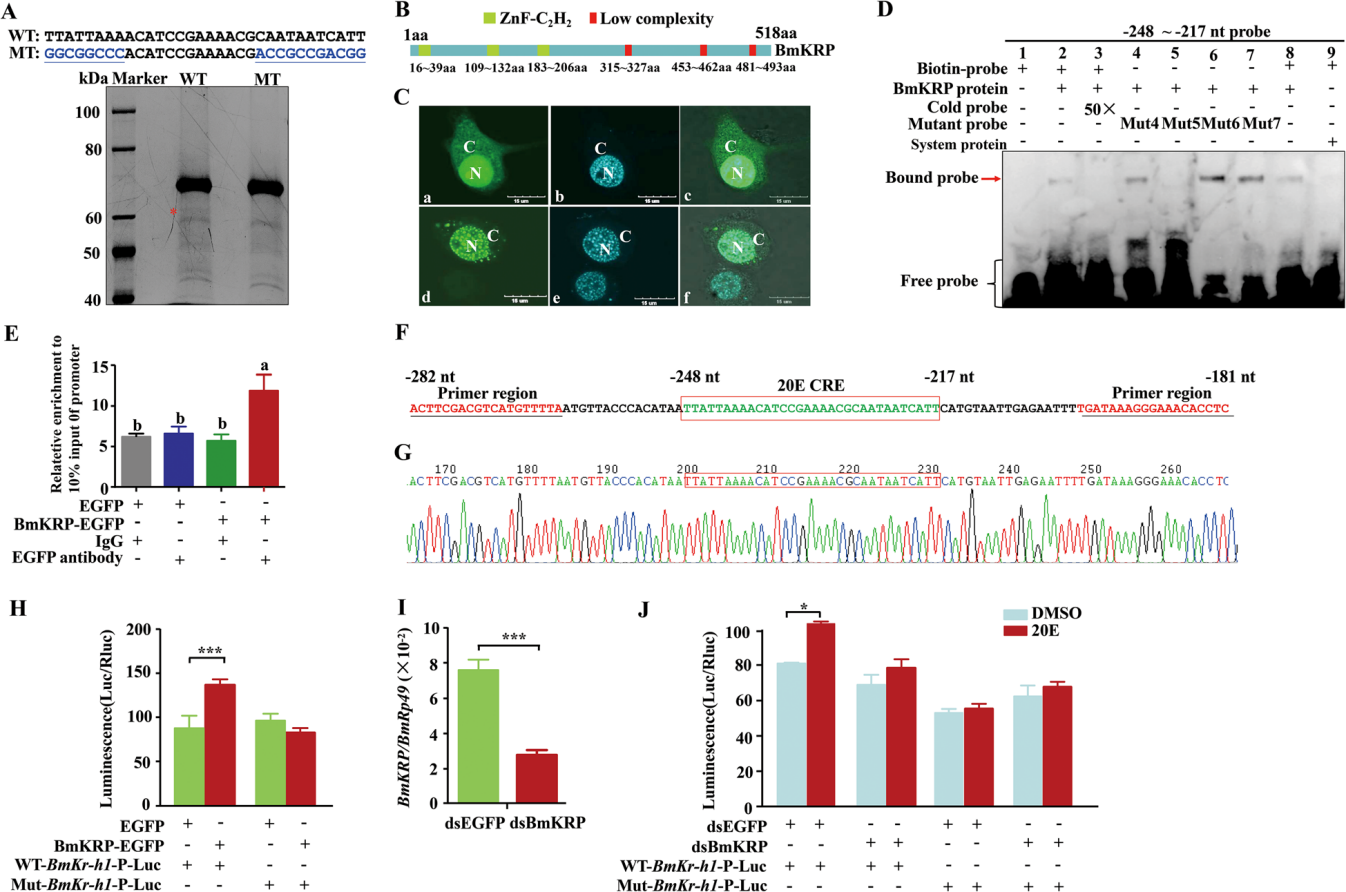
BmKRP directly binds the 20E CRE in the *BmKr-h1* promoter. **A** DNA pull-down experiment with nuclear proteins isolated from BmN cells. The oligonucleotide probes are shown in the top panel. WT, wild-type and MT, mutated ssDNA. The “*” point to the protein band that was observed in WT but not in the MT. **B** Schematic representation of the protein structure of BmKRP (Accession no. XM_004922171.3). **C** Subcellular localization of BmKRP protein in BmN cells. BmN cells were transfected with pEGFP (a–c) or BmKRP-EGFP (d–f). The bars represent 15 μm. N, nucleus; C, cytoplasm. **D** EMSA of translated recombinant BmKRP protein binding to the − 248 ~ − 217 nt probe. **E** Chromatin immunoprecipitation (ChIP) assay of expressed BmKRP-EGFP binding to the 20E CRE in the *BmKr-h1* promoter in BmN cells. The ChIP target sequence was detected by qRT-PCR. The enrichment of the promoter sequence in the immunoprecipitated DNA samples was normalized with DNA present in the 10% input material. **F** The sequence of the − 282 ~ − 181 nt region of the *BmKr-h1* promoter. The region that bound to BmKRP is boxed in red. The primer aligned regions are underlined. **G** The sequencing atlas of the enriched RT-PCR product of the ChIP assay. **H** Effect of BmKRP on luciferase activity driven by the *BmKr-h1* promoter with either the wild-type or mutant form of the 20E CRE in BmN cells. **I** *BmKRP* expression detected at 48 h after BmKRP dsRNA was transfected into BmN cells. **J** Changes in luciferase activity in BmN cells co-transfected with BmKRP dsRNA or EGFP dsRNA and the luciferase reporter vector treated by 20E. The data in **E**, **H**, **I**, and **J** are means ± SEM (*n* = 3), and the individual data values are shown in Additional file 2. Different letters above the columns indicate significant differences in luminescence at *p* < 0.05 by ANOVA. The significance of the differences between the treatment and control was statistically analyzed at *p* < 0.05 (*), *p* < 0.01 (**), and *p* < 0.001 (***) using *t* test

To identify the nuclear protein(s) that bind the 20E CRE (− 248 ~ − 217 nt) in the *BmKr-h1* promoter, a DNA pull-down assay was performed. The biotin-labeled CRE containing nucleotide fragment (TTATTAAAACATCCGAAAACGCAATAATCATT) was linked to the streptavidin-coated beads and incubated with nuclear proteins isolated from 20E-treated BmN cells. A mutated probe (GGCGGCCCACATCCGAAAACGACCGCCGACGG) that does not bind the nuclear proteins was used as a control. The protein(s) binding the probe were then separated on an SDS-PAGE gel, and the protein bands present in the purified proteins using the wild-type CRE but not in those purified with the mutant CRE (Fig. 4A) were excised and subjected to LC-MS/MS analysis. LC-MS/MS analysis identified fifteen candidate proteins (Additional file 1, Table S2). One of these proteins, BmLOC101738779 (GenBank accession no. XM_004922171.3) is a nucleic acid-binding and potential 20E CRE-binding protein and hence named BmKRP (*B. mori Kr-h1* regulatory protein). This protein consists of 518 amino acids with a predicted molecular mass of 59 kDa and a pI of 9.25, containing three ZnF-C_2_H_2_ domains at its *N*-terminal end and three low-complexity regions at the *C*-terminal end (Fig. 4B and Additional file 1, Fig. S6).

To examine subcellular localization of BmKRP, expression vectors of BmKRP-EGFP and EGFP (as a control) were constructed and used to transfect BmN cells. The localization of the expressed proteins was examined under a confocal microscope. The green fluorescence signal in the cells transfected with EGFP alone was present in both the nuclei and cytoplasm (Fig. 4C a–c), whereas the BmKRP-EGFP signal was exclusively localized to the nuclei (Fig. 4C d–f), suggesting that this protein may be involved in the regulation of *BmKr-h1* transcription in the nucleus.

To determine whether this protein binds the 20E CRE in the *BmKr-h1* promoter, the protein was translated in vitro and used in EMSA. BmKRP specifically bound the labeled − 248 ~ − 217 nt probe (Fig. 4D, lane 2 and 8), and the binding was competed off by the 50-fold excess unlabeled probe (Fig. 4D, lane 3). No such binding was found when the protein was translated from control vector DNA (Fig. 4D, lane 9). The BmKRP protein bound the mutated probes 4, 6, and 7 (Fig. 4D, lane 4, 6, and 7), but did not bind the mutated probe 5 (Fig. 4D, lane 5). These results indicate that the binding of the recombinant protein with the CRE is consistent with that of the nuclear proteins from BmN cells (Fig. 3f).

To further demonstrate whether BmKRP bound the 20E CRE in the *BmKr-h1* promoter in vivo, a ChIP assay was performed. BmKRP was expressed as an EGFP fusion protein and the antibodies that recognize EGFP were used in ChIP assay. BmN cells were transfected with BmKRP-EGFP or EGFP (as a control) for 48 h. The cells were then collected and processed for ChIP assay. The EGFP antibodies, but not IgG (negative control), precipitated and enriched the 20E CRE fragment of the *BmKr-h1* promoter in the cells transfected with the BmKRP-EGFP-expressing plasmid (Fig. 4E). The PCR-enriched 20E CRE sequence was confirmed by sequencing (Fig. 4F, G). These experiments demonstrated that BmKRP, which was localized to the nucleus (Fig. 4C d-f), binds the 20E CRE located in the − 248 ~ − 217 nt region of the *BmKr-h1* promoter.

To determine whether the binding of BmKRP to the 20E CRE influenced the *BmKr-h1* promoter transcription activity, the BmKRP-EGFP expression vector was co-transfected into BmN cells with the *BmKr-h1*-Promoter (− 248/+ 81 bp)-Luc construct. The *BmKr-h1* promoter-controlled luciferase activity increased in the BmKRP-EGFP-transfected cells but not in the control cells (Fig. 4H). To confirm the BmKRP overexpression result, BmKRP RNAi was performed. When the BmKRP expression was downregulated by dsBmKRP in BmN cells (Fig. 4I), the transcription activity of the wild-type *BmKr-h1* promoter induced by 20E disappeared compared to the control cells treated with dsEGFP (Fig. 4J). The transcription activity of the mutated *BmKr-h1* promoter did not respond to 20E challenge in either dsBmKRP- or dsEGFP-treated cells (Fig. 4J).

The developmental and 20E-induced expression patterns of *BmKRP* were then examined. qRT-PCR analysis showed that in the ovaries, the BmKRP mRNA levels were low during the early- and mid-pupal stages, but dramatically increased during the late pupal and adult stages (Additional file 1, Fig. S7A). Treatment with 20E increased the expression of *BmKRP* in the cultured ovaries dissected from 5-day-old pupae and in BmN cells (Additional file 1, Fig. S7B-D). Additionally, a potential EcRE (− 1717 ~ − 1705 nt, AGTTCAGTGACCT) was predicted in the promoter of *BmKRP* (Additional file 1, Fig. S7E) based on the consensus EcRE sequence (5′-(A/G)G(G/T)TCANTGA(C/A)C(C/T)-3′) for 20E/EcR/USP binding [33]. These results indicate that 20E induces the *BmKRP* expression by binding the EcR/USP complex which in turn interacts with the potential EcRE in the *BmKRP* promoter. BmKRP then binds the 20E CRE in the *BmKr-h1* promoter and activated its expression in the ovaries and BmN cells.

### Effects of *BmKRP* knockout on ovarian development and oogenesis

**Fig. 5:**
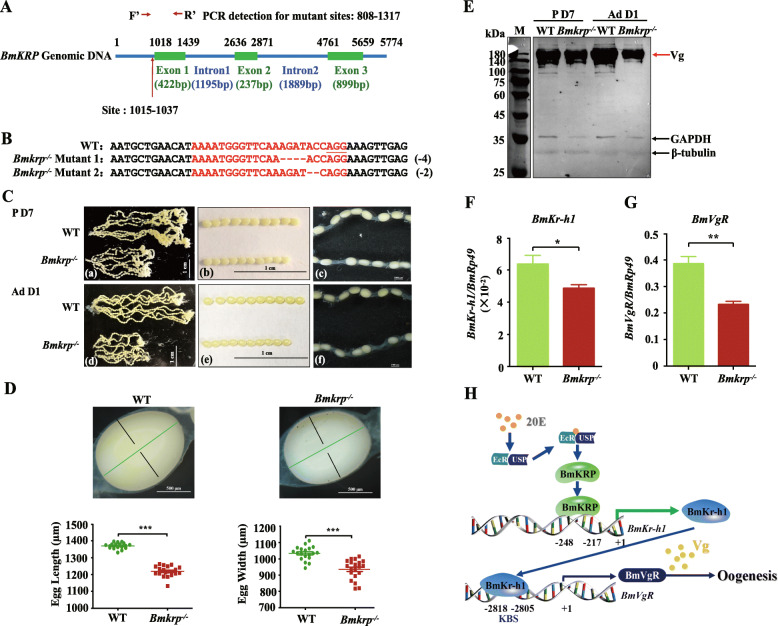
Effects of knockout of *BmKRP* by CRISPR/Cas9-mediated genome editing on ovarian development and oogenesis. **A** Schematic diagram of the sgRNA-target site. The green boxes indicate the three exons of *BmKRP*, and the blue line indicates the gene locus. The sgRNA targeting site was located on the sense strand of exon 1. F′and R′ were used for annealing the upstream and downstream regions of the targeting site. **B** Two mutant types of deletion screened from homozygous mutant silkworms in G3 generation. The wild-type sequence is shown at the top with the sgRNA target sites marked in red and PAM sequences is underlined. In the mutant sequences, deletions are shown as dashes. **C** Phenotypes of ovaries and oocytes of 7-day-old pupae (a–c) and 1-day-old adults (d–f) in G3 generation. **D** Effects of knocking down *BmKRP* on oocyte length and width of 7-day-old pupae. Green line represents the length and black line represents width. **E** Western blot analysis of BmVg protein in the oocyte. A total of 15 μg protein from the oocytes was loaded per lane and probed with anti-BmVg, anti-GAPDH, and anti-β-tubulin antibodies. qRT-PCR analysis of *BmKr-h1* (**F**) and *BmVgR* (**G**) mRNA in the oocytes of 7-day-old pupae. *Rp49* amplified from the same RNA samples was used as an internal control. **H** Schematic representation of the 20E-induced and BmKRP mediated expression of *BmKr-h1* and its physiological function in oogenesis. WT, wild-type. The data in **F** and **G** are means ± SEM (*n* = 3) and the individual data values are shown in Additional file 2. The significance of the differences between the treatment and control was statistically analyzed at *p* < 0.05 (*), *p* < 0.01 (**), and *p* < 0.001 (***) using *t* test

To determine whether BmKr-h1 promotes the oocyte development through BmKRP, the mutant *B. mori* line for *Bmkrp* was established using the CRISPR/Cas9-mediated genome editing. A 23-bp sgRNA target site located on exon 1 was identified by screening for sgRNA sites over the exons of *BmKRP* (Fig. 5A). The synthesized sgRNA was mixed with Cas9 mRNA and injected into preblastoderm embryos of the silkworm. In the third generation, the homozygous individuals were obtained and two mutant genotypes (mutant1, 4 bp deletion; mutant2, 2 bp deletion) were identified (Fig. 5B). The *Bmkrp*^*−/−*^ pupae and adults, especially females, were smaller than the wild-type (WT) animals, while the *Bmkrp*^*−/−*^ larvae were not obviously different in body size and shape from the wildtype (Additional file 1, Fig. S8B). Compared to the wild-type, the body weight, length, and width of the *Bmkrp*^*−/−*^ pupae at day 4 was lower (Additional file 1, Fig. S8C-E). The development of the ovary was incomplete and contained fewer oocytes in the *Bmkrp*^*−/−*^ pupae and adults than that in the wild-type (Fig. 5C). The number of eggs laid by the mutant females was reduced by 46% (Additional file 1, Fig. S8F-G). In addition, the length and width of oocytes in the ovarioles were reduced (Fig. 5C (b), C (e), and D). Furthermore, western blotting analysis showed that the major yolk protein Vg was reduced in the oocytes of 7-day-old pupae and 1-day-old adults, as compared to the wild-type (Fig. 5E). This is similar to the reduction of Vg in the oocytes of dsBmKr-h1-treated females (Fig. 2C). These results suggest that the depletion of *BmKRP* reduces Vg accumulation, perturbs ovarian development and arrests oogenesis.

To confirm whether *BmKRP* regulated the ovarian development and oocyte formation via *BmKr-h1* and *BmVgR*, the expression of *BmKr-h1* and *BmVgR* in the oocytes of the *Bmkrp*^−/−^ silkworm were quantified. qRT-PCR analysis showed that the expression of *BmKr-h1* and *BmVgR* decreased in the oocytes of *Bmkrp*^−/−^ pupae collected on day 7 compared to the wild-type (Fig. 5F, G), suggesting that BmKRP is a regulator of *BmKr-h1* and *BmVgR*.

## Discussion

The data presented in the present paper form lines of evidence supporting our hypothesis that 20E-induced Kr-h1 expression is mediated by a novel transcription factor BmKRP in the female reproductive system of a model lepidopteran insect *B. mori*. Our data show the following conclusions: (1) *BmKr-h1* was highly expressed in ovaries during the late pupal and adult stages when 20E titers were high and was experimentally demonstrated to be induced by 20E; (2) dsRNA knockdown of *BmKr-h1* led to arrested oogenesis via inhibiting the expression of *BmVgR*; (3) A 20E CRE was identified in the promoter of *BmKr-h1* and functionally verified using luciferase reporter assay, EMSA and DNA-ChIP; (4) A novel transcription factor BmKRP that specifically bound the 20E CRE of *BmKr-h1* promoter was shown to activate *BmKr-h1* transcription; and (5) CRISPR/Cas9-mediated knockout of *BmKRP* in female pupae suppressed the transcription of *BmKr-h1* and *BmVgR*, resulting in arrested oogenesis. These data suggest that the 20E-induced BmKRP regulates BmKr-h1 expression, which in turn activates VgR expression, facilitating Vg uptake and oogenesis.

Induction of *Kr-h1* gene by both JH and 20E has been reported in different species [16–20, 28]. The mechanisms and physiological functions of JH-induced *Kr-h1* have been well studied [15, 28]. Recent studies report that *Kr-h1* transduces the JH signal to suppress 20E primary responsive genes, such as ecdysone receptor (*EcR*), *BR-C*, and ecdysone-inducible proteins *E75* and *E93*, which subsequently lead to a lowering of 20E titer [34], and Kr-h1 inhibits the expression of steroidogenic enzyme gene *Spok* by binding the Kr-h1 binding site (KBS) to suppress the ecdysone biosynthesis [35], partially explaining the sophisticated JH-20E cross-talk. However, the physiological role and regulatory mechanism of the 20E-induced *Kr-h1* expression is not known. We investigated the physiological role and mechanisms of 20E-induced *Kr-h1* in female reproduction of *B. mori.* The developmental and 20E-induced expression studies showed that *BmKr-h1* is induced by 20E in the cultured ovaries of 5-day-old pupae and BmN cells of *B. mori* (Fig. 1), suggesting a role for the 20E-induced *BmKr-h1*. In *B. mori*, 20E-dependent oogenesis occurs almost exclusively during pupal and pharate adult stages when JH is absent and is completed before adult eclosion [36]. A high ecdysone titer was detected in the hemolymph of 3-day-old pupae [29] and in the ovaries of 7-day-old pupae in *B. mori* [30]. RNAi-mediated silencing of *BmKr-h1* in late female pupae downregulated the expression of *BmVgR* and reduced the deposition of Vg protein into the oocytes (Fig. 2), suggesting that BmKr-h1 regulates the uptake of yolk protein through BmVgR.

Vg synthesis and ovarian development are co-regulated by a complex interaction between 20E, JH and nutritional signaling pathway, in which *Kr-h1* plays critical roles [37]. In *H. armigera*, *HaKr-h1* RNAi results in atrophied ovaries with less yolk protein deposition [19]. In *L. migratoria*, *N. lugens*, *A. aegypti*, *H. armigera*, *B. dorsalis* and *S. furcifera*, depletion of *Kr-h1* prevents JH-regulated expression of Vg and oocyte maturation, ovarian development and egg production [19, 21–25]. Knocking down *Kr-h1* in female adults of *C. lectularius* does not reduce their fecundity but affects the embryonic development [27]. In *N. lugens*, silencing of both *Broad-complex* and *Kr-h1* increases the number of eggs laid by long-winged females, whereas knocking down either one of these two genes decreases the number of eggs laid [38]. The inhibitory effect of knocking down *E93* on ovarian development and egg number is partially recovered by *Kr-h1* knockdown [39]. Thus, either JH or 20E regulates *Kr-h1* expression, vitellogenesis and ovarian development in different insect species. These differences may be due to differential hormonal regulation of oogenesis and the various types of ovaries. For instance, JH regulates the reproduction in hemimetabolous insect orders, such as Orthoptera (*L. migratoria*) [21, 40, 41], Blattodea (*Blattella germanica*, *Diploptera punctata*) [42–44] and Hemiptera (*Pyrrhocoris apterus*, *C. lectularius*, *N. lugens*) [14, 22, 45]. In holometabolous coleopteran *T. castaneum*, JH is the primary hormone governing reproduction [26], but ecdysteroids are involved in oocyte maturation [46]. In lepidopterans, reproduction regulation appears to be quite different. JH has a significant role in many species, including *Manduca sexta* and *H. armigera*, but in others, such as *B. mori*, ecdysteroids are the major hormone controlling egg development [37]. In the fall armyworm *Spodoptera frugiperda*, ecdysteroids stimulate vitellogenesis, and JH promotes Vg uptake into the ovaries [47]. These results indicate the complexity in the hormonal regulation of the reproductive process in insects and both JH and 20E may contribute to different steps of the process.

The present study found that BmKr-h1 regulated the expression of *BmVgR* by directly binding its promoter, enhancing Vg uptake into oocytes. Depletion of either *BmKRP* or *BmKr-h1* suppressed the *BmVgR* expression in late female pupae (Figs. 2D and 5G), and overexpression of *BmKr-h1* increased the expression of *BmVgR* in BmN cells (Fig. 2E). BmKr-h1 specifically bound the KBS located at the − 2818 ~ − 2805 nt upstream regulatory region of *BmVgR* (Fig. 2G)*. Kr-h1* had been reported to contribute to an anti-metamorphic function of JH by binding to the KBS sequences of *E93*, *BR-C* and steroidogenic enzyme genes [3, 31, 34, 35]. Although *Kr-h1* had been found to be involved in female reproduction, the detailed molecular regulatory mechanism had not been clearly understood. Here, we reported the identification of the *Kr-h1* target gene, *BmVgR*, and the molecular mechanism of *Kr-h1* function in female reproduction. *BmKr-h1* upregulated the *BmVgR* expression by binding to the KBS in its promoter, enhancing the Vg uptake by oocytes and egg maturation. It must be noted that we could not exclude the possibility that other genes involved in the oogenesis might also be regulated by BmKr-h1 and further investigation is needed.

Because *BmKr-h1* showed a rapid response to 20E, a 20E CRE in the upstream regulatory region of the gene was expected. The − 248 ~ − 217 nt region of the *BmKr-h1* gene was identified as the 20E CRE region (Fig. 3), the sequence of which differed from EcRE identified in 20E-response genes [33]. In particular, the sequences at − 248 ~ − 241 nt (TTATTAA) and − 227 ~ − 217 nt (AATAATCATT) regions, separated by 13 bp, are required for the 20E response (Fig. 3d, f). When either one of the CREs was mutated (Mut 6 and Mut 7), the binding with BmKRP still occurred although the binding became weaker (Fig. 3f, lane 9 and 10). Similar 20E CREs, TTATTAA and AATAATCATT, which are adjacent to each other or partially overlapping, were also found in the upstream region of *Kr-h1* genes in *T. castaneum*, *A. mellifera*, *A. aegypti*, *A. pisum* and *D. melanogaster* (Additional file 1, Fig. S5), indicating that the 20E CREs of *Kr-h1* are conserved across different species. The EcRE upstream of *BmEcR-B1* is also composed of two adjacent elements (E1 and E2) with 5 bp separation, and both are required for the 20E response and the two-component structure of E1 and E2 in *EcR-B1* was conserved in lepidopteran species [48]. The above results suggest that two adjacent 20E CRE in the upstream of a gene may enable the rapid induction of gene transcription by 20E, which probably is a common mechanism in the 20E signaling pathway.

The relationship between the JH response element (JHRE) and 20E CRE in the promoter region of *BmKr-h1* is an interesting subject. The two 20E CREs are located at − 248 ~ − 241 nt and − 227 ~ − 217 nt regions, while the JHRE in the *BmKr-h1* promoter is located at the − 2165 ~ − 2025 nt region [28]. These two 20E CREs and JHRE are separated by about 1917 bps in the *BmKr-h1* promoter. How they interact or cooperate to regulate the *BmKr-h1* expression is not known yet. They might function separately or simultaneously in different tissues and stages so that *BmKr-h1* could differentially respond to JH and 20E in the regulation of molting, metamorphosis and reproduction. This is an interesting issue that should be further investigated in a future study.

Identification of proteins that participate in the regulation of *BmKr-h1* by binding the CRE is an important objective of this study. A *BmKr-h1* regulatory protein, BmKRP, was identified and demonstrated to be able to bind the CREs in the *BmKr-h1* promoter (Fig. 4). BmKRP contains three ZnF-C_2_H_2_ domains at the *N*-terminal end and is localized in the nuclei of BmN cells (Additional file 1, Fig. S6 and Fig. 4B, C). *BmKRP* had high expression levels in the ovaries during the late-pupal and adult stages and was induced by 20E in the cultured ovaries and ovarian BmN cells (Additional file 1, Fig. S7), as was *BmKr-h1* (Fig. 1) and *BmVgR* (Additional file 1, Fig. S3A-D). Overexpressing BmKRP enhanced the promoter activity of *BmKr-h1*, whereas knocking-down *BmKRP* with RNAi decreased the promoter activity of *BmKr-h1* in BmN cells (Fig. 4H–J). This evidence suggests that BmKRP in responding to 20E mediates *BmKr-h1* expression by binding its 20E CRE. It would be interesting to know how BmKRP, which has three ZnF-C_2_H_2_ domains, structurally bind the two CREs in the *BmKr-h1* promoter. In addition, knocking out *BmKRP* by CRISPR/Cas9 method also reduced the expression of *BmKr-h1* and *BmVgR* (Fig. 5F, G) and Vg deposition in the oocytes (Fig. 5E), resulting in arrested development in ovaries and oogenesis (Fig. 5C, D) that was also observed in the dsBmKr-h1-treated females (Fig. 2). These results suggest that the 20E-induced *BmKr-h1* expression is mediated by BmKRP and promotes oocyte development through *BmVgR*.

In summary, in the ovaries of late pupae, 20E induced the expression of *BmKRP*, which activated the *BmKr-h1* expression by interacting with the 20E CREs in its promoter. Then, *BmKr-h1* bound the KBS in the promoter of *BmVgR* to activate its expression. *BmVgR* facilitated the BmVg uptake by the oocytes, resulting in oocyte maturation (Fig. 5H). In this model, *BmKr-h1* is a critical player between the 20E signal and oogenesis.

## Conclusion

The induced expression of *Kr-h1* by 20E exists in a variety of insects. *BmKr-h1* was highly expressed in ovaries during the late pupal and adult stages and the expression was induced by 20E. RNAi-mediated Depletion of *BmKr-h1* in female pupae during the late pupal stage repressed the transcription of *vitellogenin receptor* (*VgR*) and reduced the Vg deposition in oocytes. Then, a 20E CRE in the promoter region of *BmKr-h1* and a novel *Kr-h1* regulatory protein (BmKRP) that binds this CRE were identified. Furthermore, in responding to 20E stimulation, BmKRP was shown to induce *BmKr-h1* expression, which in turn induced *BmVgR* expression to facilitate Vg uptake and oogenesis*.* Our data demonstrated that 20E-induced BmKRP mediated *Kr-h1* expression in the female reproduction of *B. mori*.

## Methods

### Insects, cells, and treatments

The silkworm *B. mori* strain P50 was provided by the Research and Development Center of the Sericultural Research Institute of the Academy of Agricultural Sciences of Guangdong Province, China. Larvae were reared in incubators at 25–27 °C under a photoperiod of 12 h light and 12 h dark and fed with fresh mulberry leaves. Under these conditions, larvae wandered on day 6 or 7 of the 5th instar and pupated 3 days thereafter. The pupal stage was 8 days and then emerged to adults. P0 was the moment at which larvae just shed the remnants of the larval integument, marking the beginning of the pupal stage. The ovaries in 5-day-old pupae were prepared for in vitro culture. The ovaries and oocytes were dissected, rinsed with phosphate-buffered saline (PBS), and cultured in Grace’s medium (Gibco, Grand Island, NY, USA). A BmN (*B. mori* ovaries derived) cell line was maintained at 28 °C in Grace’s Insect Medium (Gibco, Grand Island, NY, USA) and with 10% FBS (Gibco, Grand Island, NY, USA).

The cultured ovaries and BmN cells were treated with different concentrations of 20E (Sigma Co., St. Louis, MO, USA) and the ovaries or cells were collected after 4 h in culture. In the time-course experiment, the cultured ovaries or BmN cells were treated with 1 μM 20E and collected in different time points. All the samples were immediately frozen with liquid nitrogen at the end of culture and stored at − 80 °C until use for RNA extraction.

### RNA extraction and quantitation real-time PCR (qRT-PCR)

Total RNA was extracted from the collected ovaries, the cultured ovaries, oocytes or the BmN cells and first-strand cDNA was synthesized according to the instructions (TaKaRa Co. Dalian, China). The primers used were shown in Table S3 (Additional file 1). *BmKr-h1* expression levels were determined by qRT-PCR using SYBR Select Master Mix (Applied Biosystems, Austin, USA) according to the instructions and were calculated by the comparative Ct (2^- ∆∆Ct^) method [49]. All the experiments were performed for three replicates.

### *BmKr-h1* promoter cloning and site-directed mutagenesis

The upstream regulatory region (from − 2877 bp to + 81 bp) of *BmKr-h1* was amplified based on the sequence from NCBI (GenBank accession no.: BAJ05087) and cloned into pMD-18 T vector (TaKaRa Co., Dalian, China). A series of stepwise truncated fragments of the *BmKr-h1* regulatory region starting at position + 81 bp and extending to − 1877, − 877, − 473, − 431, − 374, − 326, − 291, − 248, and − 202 bp were generated by PCR amplification using the *BmKr-h1*-Promoter (− 2877/+ 81 bp) plasmid as a template and ligated to the luciferase reporter plasmid, pGL3-basic vector (Promega, Madison, WI, USA). The sequences of the forward primers and a common reverse primer are listed in Supplementary Table S3. The mutant (mutated sites from − 248 to − 202 nt) *BmKr-h1*-Promoter (− 2877/+ 81 bp) were generated with the method of overlap extension PCR using the wild-type *BmKr-h1*-Promoter (− 2877/+ 81 bp) plasmid as a template. The mutant was confirmed by DNA sequencing and then inserted to pGL3-basic vector for activity assay.

### Luciferase assay for promoter activity

Cell transfection and co-transfection were conducted when the cell density achieved 80%. To normalize the firefly luciferase activity, the renilla luciferase vector pRL-SV40 (Promega, Madison, USA) was co-transfected with each of the pGL3-derived vectors. For transfection, a mix of 50 μL containing 500 ng of the wild-type or mutated *BmKr-h1* promoter-pGL3 reporter plasmid DNA, 50 ng internal control plasmid and 1.5 μL Fugene HD transfection reagent (Promega, Madison, USA) in the Opti-MEM reduced serum medium (Invitrogen, CA, USA) was added to BmN cells in Grace medium with 0.005% DMSO (final concentration) or 1 μM 20E in 0.005% DMSO. For co-transfection, a mix of 50 μL containing 500 ng of the wild-type or mutated *BmKr-h1* promoter-pGL3 reporter plasmid DNA, 500 ng EGFP or BmKRP-EGFP plasmid DNA, 100 ng internal control plasmid (pRL-SV40 vector) and 3 μL Fugene HD transfection reagent in the Opti-MEM Reduced Serum Medium was added to the cells. The cells were cultured for 48 h at 28 °C before the promoter activity assay. The luciferase activity assay was carried out according to the instruction of the Dual-Luciferase® Reporter Assay System Kit (Promega, Madison, USA). All assays were repeated at least three times. The luciferase activity was represented as mean ± standard error (SE). Statistical significance of the luciferase activity was analyzed using the Student’s *t* test.

### DNA pull-down and liquid chromatography-tandem mass spectrometry (LC-MS/MS) analysis

Nuclear protein of BmN cells was extracted using NE-PER Nuclear and Cytoplasmic Extraction Reagents (Thermo Scientific, Waltham, USA). The protein concentration was determined using BCA Protein Assay Reagent Kit (Thermo Scientific, Waltham, USA). DNA oligonucleotide sequences of the − 248 ~ − 217 nt probe (TTATTAAAACATCCGAAAACGCAATAATCATT) and the mutated probe (GGCGGCCCACATCCGAAAACGACCGCCGACGG) were heated at 95 °C for 10 min in 50 mM Tris buffer at pH 7.5 and slowly cooled to room temperature over 4 h to generate the double-stranded probe. Double-stranded biotinylated DNA oligonucleotide (20 μg) was incubated with 100 μg streptavidin-coated Dynabeads (Life Technologies, USA) in 400 μL binding buffer (10 mM Tris, pH 7.5, 1 mM EDTA, 1 M NaCl, 0.003% NP40) for 30 min at room temperature with constant and slow rotation. After twice washing with binding buffer, the immobilized DNA was incubated for 30 min in 400 μL blocking buffer (2.5 mg/ml BSA, 10 mM HEPES, pH 7.6, 100 mM potassium glutamate, 2.5 mM DTT, 10 mM magnesium acetate, 5 mM EGTA, 3.5% glycerol with 0.003% NP40, and 5 mg/ml polyvinylpyrrolidone). Then, 100 μg of nuclear proteins were incubated with the immobilized DNA for 4 h at 4 °C in 400 μL protein binding buffer (10 mM HEPES, pH 7.6, 100 mM potassium glutamate, 80 mM KCl, 2.5 mM DTT, 10 mM magnesium acetate, 5 mM EGTA, 3.5% glycerol with 0.001% NP40, and 1 μg non-specific DNA) with constant and slow rotation. After 4 h, the DNA/protein complexes were washed three times with 400 μL washing buffer (10 mM HEPES, pH 7.6, 100 mM potassium glutamate, 2.5 mM DTT, 10 mM magnesium acetate, 5 mM EGTA, 3.5% glycerol, 0.5 mg/ml BSA, 0.05% NP40). The proteins bound to the DNA were then eluted in 20 μL SDS-PAGE sample buffer (50 mM Tris, 100 mM DTT, 2% SDS, 0.1% Bromophenol blue, 10% glycerol). The eluted proteins were subjected to 12% SDS-PAGE. The gels were stained with Coomassie Brilliant Blue R-250 for more than 4 h and distained with distaining solution (10% acetic acid, 5% ethanol, 85% water). The differentiated protein bands were excised and subjected to protein identification using LC-MS/MS in Huijun Biotechnology company (Guangzhou, China).

### Construction of ex vitro translation system

*BmKRP* open reading frame (ORF) fragment was amplified and then sub-cloned into the pF25A ICE T7 Flexi vector (Promega, Madison, USA) infusion with T7 promoter between *Sgf* I and *Pme* I restriction enzyme sites, generating a BmKRP-pF25A ICE T7 Flexi recombinant expression vector. BmKRP-pF25A ICE T7 Flexi (4 μg) was used as a template for ex vitro translation in the TNT Quick Coupled Transcription/Translation System (Promega, Madison, USA) containing 40 μL of TNT T7 Quick Master Mix and nuclease-free water of a final volume of 50 μL. The reaction was performed at 30 °C for 4 h and 2 μL of translation product was then used for EMSA assay.

### Electrophoretic mobility shift assay (EMSA) for protein-DNA binding

EMSA was conducted using the Light Shift Chemiluminescent EMSA Kit (Thermo Scientific, Waltham, USA). The wild-type and mutant oligonucleotides of − 248 ~ − 217 nt and − 234 ~ − 203 nt probes (Fig. 3d) were labeled with biotin at the 5′ end and heated at 95 °C for 10 min in 50 mM Tris-acetate buffer at pH 4.1 and slowly cooled to room temperature. The biotin-labeled oligonucleotides were synthesized by Invitrogen (Shanghai, China). Binding reactions were performed according to the instruction of the EMSA Kit. Briefly, reactions were conducted in a 20 μL mix containing (1 × binding buffer, 2.5% glycerol, 0.05% NP-40, 50 mM KCl, 5 mM MgCl_2_, 4 mM EDTA, 1 μg poly dI-dC, 2 μg nuclear proteins or 2 μL of translated product BmKRP and 20 fmol of a biotinylated end-labeled probe) at room temperature for 20 min. For the competition assay, cold probes (un-biotinylated) were added to the binding reaction. The samples were then separated in 6% polyacrylamide gels on the ice at 100 V for 1.5 h. After electrophoresis, the gels were blotted onto a Nylon positively charged membrane (Amersham Biosciences, Fairfield, USA). The membranes were then developed using the Light Shift Chemiluminescent EMSA kit according to the manufacturer’s protocol.

### Subcellular localization of BmKRP protein

For subcellular localization study of BmKRP, the ORF DNA fragment of *BmKRP* was PCR amplified with a FLAG tag and a His-tag between *Kpn* I and *Bam*H I with forward primer: 5′- GGTACCGATGGATTACAAGGATGACGACGATAAGGGTTCAAAGATACCAG-3′ and reverse primer: 5′- GGATCCCGGTGATGATGATGATGATGAATTTTTATAACCATA-3′ and cloned into pEGFP-N1 plasmid vector (Clontech Laboratories Inc., Mountain View, CA, USA), generating a recombinant plasmid vector, BmKRP-EGFP. BmKRP-EGFP was transfected into BmN cells. BmKRP-EGFP protein was localized by observing the green fluorescence signal under a laser confocal microscope (Carl Zeiss AG, Oberkochen, Germany) at 24 h after transfection. The vector expressing EGFP alone was used as control. The nuclei were counterstained by DAPI.

### Chromatin immunoprecipitation (ChIP) assay

ChIP assay was conducted to detect the binding of BmKRP with the 20E *cis*-response element of the *BmKr-h1* promoter in BmN cells. Cells were crosslinked with 1% formaldehyde for 10 min at room temperature after being transfected with BmKRP-EGFP or EGFP (control) plasmids for 48 h. Glycine was added to terminate the fixation and the cells were washed twice with one media volume of ice-cold PBS. Cells in 1 mL of ice-cold PBS and 10 μL Halt™ Protease Inhibitor Cocktail (Thermo Scientific, Waltham, USA) were collected by scraping. The cells were centrifuged at 3000*g* for 5 min and the cell pellet was homogenized with extraction buffer containing protease/phosphatase inhibitors. Nuclei were collected by centrifuged at 9000*g* for 3 min and digested by MNase and chromatin was obtained by sonication on ice with several pulses to break nuclear membrane and incubated for 20 s on ice between pulses and then centrifuged at 9000*g* for 5 min. Immunoprecipitation was performed following the manufacturer’s instruction of Pierce™ Magnetic ChIP Kit (Thermo Scientific, Waltham, USA). Ten micrograms of either rabbit anti-EGFP antibody or normal rabbit IgG (Thermo Scientific, Waltham, USA) were used and IP reactions were incubated overnight at 4 °C with constant mixing. DNA/protein/antibody complex was purified by incubating with ChIP Grade Protein A/G Magnetic Beads for 2 h at 4 °C with mixing. Immunoprecipitated genomic DNA fragments were amplified by qRT-PCR with primers (Table S3). The length of the product was 101 bp. The specificity of the primers was examined using Primer-blast (http://www.ncbi.nlm.nih.gov/tools/primer-blast/). For qRT-PCR, the SYBR Green Kit was used according to the manufacturer’s instruction (TaKaRa, Dalian, China) and was performed at the following conditions: SYBR Premix Ex Taq (2×): 10 μL in 20 μL reaction volume, the primers concentrations: 0.4 μL (10 μM), the immunoprecipitated DNA samples: 4 μL. The mix was incubated at 95 °C for 10 s, followed by 40 cycles at 95 °C for 5 s, 60 °C for 30 s using ABI7300 fluorescence quantitative PCR system. The enrichment of the promoter sequence in the immunoprecipitated DNA samples was normalized with DNA present in the 10% input material analyzed using the comparative Ct (2^- ∆∆Ct^) method [49]. The PCR products of the enriched promoter were sequenced for confirmation.

### Generation of *Bmkrp*^*−/−*^ mutants by CRISPR/Cas9

A 23-bp sgRNA targeting site at exon I of *BmKRP* was identified (SI Appendix, Fig. 5A) according to the GGN19GG rule [50, 51]. The sgRNA DNA template was synthesized by PCR with Q5® High-Fidelity DNA Polymerase (NEB, Beijing, China), the oligonucleotide (*BmKRP*-sgF1) that encoded T7 polymerase binding site, sgRNA targeting sequence, and overlap sequence was annealed to a common oligonucleotide that encoded the remainder of the sgRNA sequence (sgRNA-R) (Table S3). The reaction conditions were as follows: 98 °C for 2 min, 35 cycles of 94 °C for 10 s, 55 °C for 30 s, and 72 °C for 2 min, followed by a final extension period of 72 °C for 10 min. The sgRNA was synthesized based on the DNA template in vitro with MAXIscript® T7 Kit (Ambion, Austin, TX, USA). The Cas9 gene template was provided by the Shanghai Institute of Plant Physiology and Ecology (Shanghai, China). Cas9 mRNA was prepared using the mMESSAGE mMACHINE® T7 kit (Ambion, Austin, USA).

The fertilized eggs were collected and injected within 6 h after oviposition. A mix of Cas9 mRNA (1000 ng/μL) and sgRNA (1000 ng/μL) was injected into the P50 embryos (about 12 nL/egg) using a micro-injector (FemtoJet®, Germany). The injected eggs were incubated at 25 °C for 9–10 days until hatching. The hatched animals were G0 individuals. Genomic DNA was extracted from the larvae-sloughs of G0 individuals for genotyping by sequencing and the multiple peaks in chromatograms of the PCR-product sequencing at the region flanking the target position were indicatives of mosaic mutations. Individuals with such mosaic mutation genotypes were used to mate with the wild-type or pair-mating to produce G1 generation offspring populations. The mosaic mutation genotypes of G1 generation were shown in SI Appendix, Fig. S8A. Two mutants with 4 bp and 2 bp deletion were selected for pair-mating to produce G2 generation, respectively. In G3 generation, the homozygous mutants with shift-frame mutation (4 bp deletion and 2 bp deletion) were identified and used for phenotypic observation.

### Genomic DNA extraction and mutagenesis analysis

Genomic PCR, followed by sequencing, was carried out to identify the *Bmkrp* mutant alleles generated by the CRISPR/Cas9 system. Genomic DNA was extracted by TIANamp Blood DNA Kit (Tiangen Biotech, Beijing, China). The PCR conditions were as follows: 94 °C for 2 min, 35 cycles of 94 °C for 30 s, 55 °C for 45 s, and 72 °C for 30 s, followed by a final extension period of 72 °C for 10 min. The amplified fragments were cloned into a pMD19-T Simple Vector (TaKARA, Dalian, China). The primers designed to detect mutagenesis in targeted sites are shown in Table S3.

### Analysis of 20E CRE in *Kr-h1* homologs and Kr-h1 binding site in *BmVgR* promoter

Genomic databases of *T. castaneum*, *A. mellifera*, *A. aegypti, Acyrthosiphon pisum*, and *Drosophila melanogaster* were searched for *Kr-h1* homologs with the tblastn program (http://blast.ncbi.nlm.nih.gov/) by using the *BmKr-h1* sequence (GenBank accession no.: BAJ05087) as a query. The 20E CRE (5′-TTATTAA-3′) and (5′-TTATTAA-3′) located in the *BmKr-h1* promoter were searched in the 5′-flanking regions of *Kr-h*1 in *T. castaneum*, *A. mellifera*, *A. aegypti, A. pisum*, and *D. melanogaster.* The *BmVgR* promoter sequence was used to identify the putative Kr-h1 binding site (KBS) in the 5′-flanking regions by comparing the KBS in the promoters of *BmE93* [3], BmBR-C [31], and steroidogenic enzyme genes [35].

### RNA interference (RNAi)

For RNAi of *BmKRP* in BmN cells, a 431-bp unique fragment between 170 and 600 nt in the ORF of *BmKRP* was used as a template for synthesizing gene-specific dsRNA from the linearized template by using the T7 RiboMAXTM Express RNAi System (Promega, Madison, USA). Total RNA extraction and reverse transcription were conducted as above mentioned. RT-PCR was performed using specific primers designed based on the *BmKRP* cDNA to detect the knockdown efficiency. To detect the effect of *BmKRP* on *BmKr-h1* promoter activity when *BmKRP* was knocked down, a 50-μL mixture containing 2.5 μg *BmKRP* dsRNA or EGFP dsRNA in 5-μL Fugene HD transfection reagent in the Opti-MEM Reduced Serum Medium (Invitrogen, CA, USA) was added to cells. After 24 h dsRNA transfection, 50 μL mixture containing 0.5 μg of the wild-type or mutated *BmKr-h1*-Promoter (− 2877/+ 81 bp)-pGL3 reporter plasmid DNA, 50 ng internal control plasmid (pRL-SV40 vector), and 1.5-μL Fugene HD transfection reagent in the Opti-MEM Reduced Serum Medium was added to cells and 1 μM 20E was added to the cells at the final concentration at 6 h post second transfection. After culture for 24 h at 28 °C, the cells were collected for the promoter activity assay.

For RNAi of *BmKr-h1* in *B. mori*, a 533 bp unique fragment between 1 and 532 nt in the ORF of *BmKr-h1* was used as a template for synthesizing gene-specific dsRNA from the linearized template by using the T7 RiboMAXTM Express RNAi System (Promega, Madison, USA). Pupae at day 6 were used for dsRNA injection. The pupae were anesthetized on ice for 5 min prior to microinjection. Fifteen micrograms dsRNA per pupa was injected into the middle ventral segment, and three replicates of 70–100 pupae each were injected for each of the treatment groups. The epidermis, wing, testis, ovary, and egg were collected for RNA analysis. Each treatment sample included at least three individual larvae and was repeated three times.

### Western blotting

Total proteins were isolated from ovaries during the pupal stage and the dsRNA treated pupae or oocytes at day 7 of wild-type and *Bmkrp*^*−/*−^ pupae and day 1 of wild-type and *Bmkrp*^*−/*−^ adult. Fifteen mircograms of proteins from the ovaries or oocytes was mixed with 5× loading buffer (250 mM Tris-HCl pH 6.8, 10% SDS, 0.5% bromophenol blue, 50% glycerol, 5% 2-ME) and separated on 12% SDS-PAGE and transferred to nitrocellulose membranes. The membranes were washed in Tris-buffered saline-Tween 20 (TBST) for 1 h with buffer change every 20 min to remove SDS, blocked with 3% (wt/vol) BSA in TBST at 37 °C for 2 h. The membranes were washed in TBST for three times with each wash for 10 min and then incubated with anti-BmVg antibody with a gentle rocking at 30 °C for 1 h. After washing with TBST, the membranes were incubated with the second antibody alkaline phosphatase (AP)-conjugated goat anti-rabbit IgG with a gentle rocking at 30 °C for 1 h. Anti-BmVg antibodies were diluted to 1:5000 in 1% (wt/vol) BSA in TBST (150 mM NaCl, 0.05% Tween20, 20 mM Tris-HCl, pH 8.0), and the second antibodies were diluted to 1:10,000 (Boster Biological Technology Co. Ltd.) in the same buffer. The mouse antibody of GAPDH or β-tubulin was used as an internal reference.

### Statistical analysis

Statistical analysis was performed using Student’s *t* test and ANOVA analysis. Data is presented as mean ± SD of three independent biological replicates and *p* < 0.05 was considered as significant. Different letters above the columns indicate significance in the group difference at *p* < 0.05*, *p* < 0.01**, and *p* < 0.001***.

## Supplementary Information

**Additional file 1: Table S1.** Statistics of phenotypes and reproduction of silkworm after RNAi. **Table S2.** Candidates of the 20E *cis*-response element binding proteins by LC-MS/MS. **Table S3.** Primers used in this study. **Figure S1.** qRT-PCR analysis of the expression and induced expression of *BmKr-h1β* by 20E in cultured ovaries and BmN cells. **Figure S2.** Development of eggs oviposited from the dsRNA-treated adults. **Figure S3.** Developmental and 20E-induced expression patterns of BmVg and *BmVgR* in the ovaries and BmN cells. **Figure S4.** EMSA of the binding of the nuclear protein isolated from BmN cells overexpressing BmKr-h1-EGFP with the KBS1 and KBS2 in the *BmVgR* promoter. **Figure S5.** Alignment of the putative 20E cis-response elements in the upstream of *Kr-h1* in other insect species. **Figure S6.** Nucleotide and deduced amino acid sequences of *BmKRP* in *B. mori*. **Figure S7.** Developmental and 20E-induced expression patterns of *BmKRP* in the ovaries and BmN cells. **Figure S8.** Cas9/sgRNA mediated gene editing of *BmKRP* in the silkworm.

**Additional file 2.** The individual data values for Figs. 1, 3a-c, Fig. 4E-J, Fig. 5D, F-G, Fig. S3A-S3D and Fig. S7A-S7D.

## Data Availability

All data generated or analyzed during this study are included in this published article and its supplementary information files. The data sets used and/or analyzed during the current study are also available from the corresponding author on reasonable request. Raw data can be found in Additional file 2.

## References

[1] Jindra M, Palli SR, Riddiford LM (2013). The juvenile hormone signaling pathway in insect development. Annu Rev Entomol.

[2] Belles X, Santos CG (2014). The MEKRE93 (Methoprene tolerant-Krüppel homolog 1-E93) pathway in the regulation of insect metamorphosis, and the homology of the pupal stage. Insect Biochem Mol Biol.

[3] Kayukawa T, Jouraku A, Ito Y, Shinoda T (2017). Molecular mechanism underlying juvenile hormone-mediated repression of precocious larval-adult metamorphosis. Proc Natl Acad Sci U S A.

[4] Raikhel AS, Brown MR, Belles X, Gilbert L, Iatrou K, Gill SS (2005). Hormonal control of reproductive processes. Comp Mol Insect Sci Vol. 3, Endocrinol.

[5] Swevers L, Iatrou K. Ecdysteroids and ecdysteroid signaling pathways during insect oogenesis. In Ecdysone: Structures and Functions ed. G. Smagghe, Dordrecht, Neth.: Springer. 2009; pp.127–64.

[6] Swevers L (2019). An update on ecdysone signaling during insect oogenesis. Curr Opin Insect Sci.

[7] Santos CG, Humann FC, Hartfelder K (2019). Juvenile hormone signaling in insect oogenesis. Curr Opin Insect Sci..

[8] Arrese EL, Soulages JL (2010). Insect fat body: energy, metabolism, and regulation. Annu Rev Entomol.

[9] Tufail M, Takeda M (2009). Insect vitellogenin/lipophorin receptors: molecular structures, role in oogenesis, and regulatory mechanisms. J Insect Physiol.

[10] Minakuchi C, Zhou X, Riddiford LM (2008). Krüppel homolog 1 (Kr-h1) mediates juvenile hormone action during metamorphosis of *Drosophila melanogaster*. Mech Dev.

[11] Minakuchi C, Namiki T, Shinoda T (2009). Krüppel homolog1, an early juvenile hormone response gene downstream of Methoprene-tolerant, mediates its anti-metamorphic action in the red flour beetle *Tribolium castaneum*. Dev Biol.

[12] Konopova B, Smykal V, Jindra M (2011). Common and distinct roles of juvenile hormone signaling genes in metamorphosis of holometabolous and hemimetabolous insects. Plos One.

[13] Lozano J, Belles X (2011). Conserved repressive function of *Krüppel homolog 1* on insect metamorphosis in hemimetabolous and holometabolous species. Sci Rep.

[14] Smykal V, Bajgar A, Provaznik J, Fexova S, Buricova M, Takaki K, Hodkova M, Jindra M, Dolezel D (2014). Juvenile hormone signaling during reproduction and development of the linden bug, *Pyrrhocoris apterus*. Insect Biochem Mol Biol.

[15] Kayukawa T, Murata M, Kobayashi I, Muramatsu D, Okada C, Uchino K, Sezutsu H, Kiuchi M, Tamura T, Hiruma K, Ishikawa Y, Shinoda T (2014). Hormonal regulation and developmental role of Krüppel homolog 1, a repressor of metamorphosis, in the silkworm *Bombyx mori*. Dev Biol.

[16] Pecasse F, Ruiz C, Richards G, Beck Y (2000). Krüppel-homolog, a stage-specific modulator of the prepupal ecdysone response, is essential for *Drosophila* metamorphosis. Dev Biol.

[17] Beckstead RB, Lam G, Thummel CS (2005). The genomic response to 20-hydroxyecdysone at the onset of *Drosophila* metamorphosis. Genome Biol.

[18] Xu J, Roy A, Palli SR (2018). Creb-binding protein plays key roles in juvenile hormone action in the red flour beetle, *Tribolium castaneum*. Sci Rep.

[19] Zhang WN, Ma L, Liu C, Chen L, Xiao HJ, Liang GM (2018). Dissecting the role of Krüppel homolog 1 in the metamorphosis and female reproduction of the cotton bollworm, *Helicoverpa armigera*. Insect Mol Biol.

[20] Wang HB, Ali SM, Moriyama M, Iwanaga M, Kawasaki H (2012). 20-hydroxyecdysone and juvenile hormone analog prevent precocious metamorphosis in recessive trimolter mutants of *Bombyx mori*. Insect Biochem Mol Biol.

[21] Song JS, Wu ZX, Wang ZM, Deng S, Zhou ST (2014). Kruppel-homolog 1 mediates juvenile hormone action to promote vitellogenesis and oocyte maturation in the migratory locust. Insect Biochem Mol Biol.

[22] Lin XD, Yao Y, Wang B (2015). Methoprene-tolerant (Met) and *Krüpple*-homologue 1 (Kr-h1) are required for ovariole development and egg maturation in the brown plant hopper. Sci Rep.

[23] Ojani R, Fu X, Ahmed T, Liu P, Zhu J (2018). Krüpple-homologue 1 acts as a repressor and an activator in the transcriptional response to juvenile hormone in adult mosquitoes. Insect Mol Biol.

[24] Yue Y, Yang RL, Wang WP, Zhou QH, Chen EH, Yuan GR, Wang JJ, Dou W (2018). Involvement of Met and Kr-h1 in JH-mediated reproduction of female *Bactrocera dorsalis* (Hendel). Front Physiol.

[25] Hu K, Tian P, Yang L, Tang Y, Qiu L, He HL, Ding WB, Li YZ (2020). Molecular characterization of the *Krüppel-homolog 1* and its role in ovarian development in *Sogatella furcifera* (Hemiptera: Delphacidae). Mol Biol Rep.

[26] Parthasarathy R, Sun Z, Bai H, Palli SR (2020). Juvenile hormone regulation of vitellogenin synthesis in the red four beetle, *Tribolium castaneum*. Insect Biochem Mol Biol.

[27] Gujar H, Palli SR (2016). Juvenile hormone regulation of female reproduction in the common bed bug, *Cimex lectularius*. Sci Rep.

[28] Kayukawa T, Minakuchi C, Namiki T, Togawa T, Yoshiyama M, Kamimura M, Mita K, Imanishi S, Kiuchi M, Ishikawa Y, Shinoda T (2012). Transcriptional regulation of juvenile hormone-mediated induction of *Krüppel homolog 1*, a repressor of insect metamorphosis. Proc Natl Acad Sci U S A.

[29] Gu SH, Chow YS (2005). Analysis of ecdysteroidogenic activity of the prothoracic glands during the last larval instar of the silkworm, *Bombyx mori*. Insect Biochem Mol Biol.

[30] Ohnishi E, Chatani F (1997). Biosynthesis of ecdysone in the isolated abdomen of the silkworm, *Bombyx mori*. Dev Growth Differ.

[31] Kayukawa T, Nagamine K, Ito Y, Nishita Y, Ishikawa Y, Shinoda T (2016). Krüppel homolog 1 inhibits insect metamorphosis via direct transcriptional repression of broad-complex, a pupal specifier gene. J Biol Chem.

[32] Zhu ZD, Hu QH, Tong CM, Yang HG, Zheng SC, Feng QL, Deng HM. Transcriptomic analysis revealed the regulation network of *BmKrüppel homolog1* in the oocyte development of *Bombyx mori*. Insect Sci. 2020. 10.1111/1744-7917.12747. 10.1111/1744-7917.12747 32283000

[33] Antoniewski C, Laval M, Lepesant JA (1993). Structural features critical to the activity of an ecdysone receptor binding site. Insect Biochem Mol Biol.

[34] Liu S, Li K, Gao Y, Liu X, Chen W, Ge W, Feng Q, Palli SR, Li S (2018). Antagonistic actions of juvenile hormone and 20-hydroxyecdysone within the ring gland determine developmental transitions in *Drosophila*. Proc Natl Acad Sci U S A.

[35] Zhang T, Song W, Li Z, Qian W, Wei L, Yang Y, Wang W, Zhou X, Meng M, Peng J, Xia Q, Perrimon N, Cheng D (2018). Krüppel homolog 1 represses insect ecdysone biosynthesis by directly inhibiting the transcription of steroidogenic enzymes. Proc Natl Acad Sci U S A.

[36] Tsuchida K, Nagata M, Suzuki A (1987). Hormonal control of ovarian development in the silkworm, *Bombyx mori*. Arch Insect Biochem Biophys.

[37] Roy S, Saha TT, Zou Z, Raikhel AS (2018). Regulatory pathways controlling female insect reproduction. Annu Rev Entomol.

[38] Jiang J, Xu Y, Lin X (2017). Role of *Broad-Complex (Br*) and *Krüppel homolog 1 (Kr-h1*) in the ovary development of *Nilaparvata lugens*. Front Physiol.

[39] Mao Y, Li Y, Gao H, Lin X (2019). The direct interaction between E93 and Kr-h1 mediated their antagonistic effect on ovary development of the brown planthopper. Int J Mol Sci.

[40] Glinka A, Wyatt G (1996). Juvenile hormone activation of gene transcription in locust fat body. Insect Biochem Mol Biol.

[41] Luo M, Li D, Wang Z, Guo W, Kang L, Zhou S (2017). Juvenile hormone differentially regulates two Grp78 genes encoding protein chaperones required for insect fat body cell homeostasis and vitellogenesis. J Biol Chem.

[42] Comas D, Piulachs MD, Belles X (1999). Fast induction of vitellogenin gene expression by juvenile hormone III in the cockroach *Blattella germanica* (L.) (Dictyoptera, Blattellidae). Insect Biochem Mol Biol.

[43] Comas D, Piulachs MD, Belles X (2001). Induction of vitellogenin gene transcription in vitro by juvenile hormone in *Blattella germanica*. Mol Cell Endocrinol.

[44] Marchal E, Hult EF, Huang J, Pang Z, Stay B, Tobe SS (2014). Methoprene-tolerant (Met) knockdown in the adult female cockroach, *Diploptera punctata*, completely inhibits ovarian development. PLoS One.

[45] Lu K, Chen X, Liu WT, Zhang XY, Chen MX, Zhou Q (2016). Nutritional signaling regulates vitellogenin synthesis and egg development through juvenile hormone in *Nilaparvata lugens* (Stål). Int J Mol Sci.

[46] Parthasarathy R, Sheng Z, Sun Z, Palli SR (2010). Ecdysteroid regulation of ovarian growth and oocyte maturation in the red flour beetle, *Tribolium castaneum*. Insect Biochem Mol Biol..

[47] Sorge D, Nauen R, Range S, Hoffmann KH (2000). Regulation of vitellogenesis in the fall armyworm, *Spodoptera frugiperda* (Lepidoptera: Noctuidae). J Insect Physiol.

[48] Shirai H, Kamimura M, Yamaguchi J, Imanishi S, Kojima T, Fujiwara H (2012). Two adjacent *cis*-regulatory elements are required for ecdysone response of *ecdysone receptor (EcR) B1* transcription. Plos One.

[49] Livak KJ, Schmittgen TD (2001). Analysis of relative gene expression data using real time quantitative PCR and the 2^(-ΔΔC(T))^ method. Methods..

[50] Fu YF, Sander JD, Reyon D, Cascio VM, Joung JK (2014). Improving CRISPR-Cas nuclease specificity using truncated guide RNAs. Nat Biotechnol.

[51] Wang Y, Li Z, Xu J, Zeng B, Ling L, You L, Chen Y, Huang Y, Tan A (2013). The CRISPR/Cas system mediates efficient genome engineering in *Bombyx mori*. Cell Res.

