# Supplementary material for: 20E-mediated regulation of BmKr-h1 by BmKRP promotes oocyte maturation
Source: BMC Biol. 2021 Feb 25;19:39. doi: 10.1186/s12915-021-00952-2 (PMC7905918; doi:10.1186/s12915-021-00952-2)
Supplement: Supplementary file 1 — Additional file 1: Table S1. Statistics of phenotypes and reproduction of silkworm after RNAi. Table S2. Candidates of the 20E cis-response element binding proteins by LC-MS/MS. Table S3. Primers used in this study. Figure S1. qRT-PCR analysis of the expression and induced expression of BmKr-h1β by 20E in cultured ovaries and BmN cells. Figure S2. Development of eggs oviposited from the dsRNA-treated adults. Figure S3. Developmental and 20E-induced expression patterns of BmVg and BmVgR in the ovaries and BmN cells. Figure S4. EMSA of the binding of the nuclear protein isolated from BmN cells overexpressing BmKr-h1-EGFP with the KBS1 and KBS2 in the BmVgR promoter. Figure S5. Alignment of the putative 20E cis-response elements in the upstream of Kr-h1 in other insect species. Figure S6. Nucleotide and deduced amino acid sequences of BmKRP in B. mori. Figure S7. Developmental and 20E-induced expression patterns of BmKRP in the ovaries and BmN cells. Figure S8. Cas9/sgRNA mediated gene editing of BmKRP in the silkworm. [file 12915_2021_952_MOESM1_ESM.docx]

**20E-mediated regulation of *BmKr-h1* by BmKRP promotes oocyte maturation**

Zidan Zhu^1^, Chunmei Tong^1^, Binbin Qiu^1^, Hongguang Yang^1^, Jiahui Xu^1^, Sichun Zheng^1^, Qisheng Song^2^, Qili Feng^1^*, Huimin Deng^1^*

^1^Guangdong Key Laboratory of Insect Developmental Biology and Applied Technology, Guangzhou Key Laboratory of Insect Development Regulation and Application Research, Institute of Insect Science and Technology & School of Life Sciences, South China Normal University, Guangzhou 510631, China; ^2^Division of Plant Sciences, University of Missouri, Columbia, MO, 65211, USA.

***Corresponding authors:** Qili Feng, School of Life Sciences, South China Normal University, Guangzhou, China, 510631. E-mail address: qlfeng@scnu.edu.cn

Huimin Deng, School of Life Sciences, South China Normal University, Guangzhou, China, 510631. E-mail address: denghuiminmin@163.com

Running title: *BmKr-h1* function in oogenesis.

**Supplementary Information**

**Additional file, Table S1. Statistics of phenotypes and reproduction of silkworm after RNAi**

| dsRNA | *EGFP* | *BmKr-h1* |
| --- | --- | --- |
| Total treated pupae | 85 | 96 |
| Adults (%) | 94.1 | 94.8 |
| Adults with abnormal wings (%) | 10.0 | 12.1 |
| Number of eggs | 312.4 ± 64.8 | 317.5 ± 64.0 |
| Number of eggs hatched (%) | 92.6 ± 5.1 | 86.0 ± 4.6* |
| Abnormal eggs (%) | 0.2 ± 0.6 | 13.1 ± 2.8*** |
| Abnormal silkworm (%) | 15.4 | 78.3 *** |

* indicates that the differences are significant at *p*<0.05 by *t*-test. *** indicates that the differences are significant at *p*<0.001 by *t*-test.

**Additional file, Table S2. Candidates of the 20E *cis*-response element binding proteins by LC-MS/MS**

| Protein | Molecular function | Molecular Weight | pI |
| --- | --- | --- | --- |
| LOC101741707 | ATP binding, protein refolding | 61 kDa | 5.51 |
| Yellow-16 |  | 52 kDa | 5.18 |
| Lysozyme |  | 59 kDa | 9.4 |
| CYP303A1 | heme binding, iron ion binding, oxidoreductase activity | 57 kDa | 8.57 |
| eIF4AIII | helicase activity, ATP binding, nucleic acid binding | 46 kDa | 5.6 |
| LOC101738711 | regulation of transcription, DNA-templated | 60 kDa | 7.93 |
| ATP synthase subunit alpha | ATP synthase | 60 kDa | 9.21 |
| LOC101738779 | nucleic acid binding | 59 kDa | 9.25 |
| Elongation factor 1-alpha | GTPase activity, GTP binding, translation elongation factor activity | 50 kDa | 9.21 |
| Dead box protein 2 | helicase activity, ATP binding, nucleic acid binding | 60 kDa | 9.15 |
| LOC101738850 | protein kinase | 58 kDa | 9.8 |
| LOC101741707 | ATP binding, protein refolding | 61 kDa | 5.51 |
| LOC101744752 | RNA binding, pseudouridine synthase | 57 kDa | 9.28 |
| LOC101739411 | histone modification, transcription elongation from RNA polymerase II promoter | 62 kDa | 9.75 |
| LOC101737105 | Hydrolase, hydrolase activity, hydrolyzing O-glycosyl compounds | 57 kDa | 6.58 |

**Additional file, Table S3. Primers used in this study**

| Primer | Forward (5’-3’) | Reverse (5’-3’) |
| --- | --- | --- |
| *BmKr-h1*-p-1877-pGL3 | CTCGAGTATTTAATTATTAGAGTACG | AAGCTTAATGTTGGCGTAGGTTGTGATTC |
| *BmKr-h1*-p-877-pGL3 | CTCGAGTAAATTAATGAGTGTGTGAG | AAGCTTAATGTTGGCGTAGGTTGTGATTC |
| *BmKr-h*1-p-473-pGL3 | CTCGAGGGTGGTCACGACCCTCA | AAGCTTAATGTTGGCGTAGGTTGTGATTC |
| *BmKr-h1*-p-431-pGL3 | CTCGAGGAAGTGATTTAGCATAAATAAATGA | AAGCTTAATGTTGGCGTAGGTTGTGATTC |
| *BmKr-h1*-p-374-pGL3 | CTCGAGTGCTCTGTTCAGCTTATTTCTGCCG | AAGCTTAATGTTGGCGTAGGTTGTGATTC |
| *BmKr-h1*-p-326-pGL3 | CTCGAGTAAGGGTGGATGGTGAT | AAGCTTAATGTTGGCGTAGGTTGTGATTC |
| *BmKr-h1*-p-291-pGL3 | CTCGAGAAAATTGAAACTTCGACGTCATGTTT | AAGCTTAATGTTGGCGTAGGTTGTGATTC |
| *BmKr-h1*-p-248-pGL3 | CTCGAGTTATTAAAACATCCGAAAACGCAAT | AAGCTTAATGTTGGCGTAGGTTGTGATTC |
| *BmKr-h1*-p-202-pGL3 | CTCGAGTTTTGATAAAGGGAAACACC | AAGCTTAATGTTGGCGTAGGTTGTGATTC |
| *BmKr-h1*-p-2877-mut-pGL3-1 | CTCGAGATATGTACTACATTACACTTTGACTT | TTATCAAAAGGAGACCGGCACGTCCGTCGGCGGTATGGGGATTCGTGGGGCCGCCTTATGTGGGTAACATTAA |
| *BmKr-h1*-p-2877-mut-pGL3-2 | TTAATGTTACCCACATAAGGCGGCCCCACGAATCCCCATACCGCCGACGGACGTGCCGGTCTCCTTTTGATAA | AAGCTTAATGTTGGCGTAGGTTGTGATTC |
| *BmdsKRP*-1 | TAATACGACTCACTATAGGGGAAGCTTATACCCCATAAAAAGGT | TAGTTCGTACAAATGTAAAGTATTG |
| *BmdsKRP*-2 | GGAAGCTTATACCCCATAAAAAGGT | TAATACGACTCACTATAGGTAGTTCGTACAAATGTAAAGTATTG |
| *BmKr-h1* qRT-PCR | CACTTCGCATCCAAATCATC | GATCGTGCGTGTGCTGTAAG |
| *BmKRP* qRT-PCR | ACCAGAATAGGGCAGTTCAAG | CTTTGGTTGAAGACTTGGGC |
| *BmVgR* qRT-PCR | ACGACAGAGAACAGCAACAG | AAGCAACAGGAGATACCGTG |
| *BmRp49* qRT-PCR | CAGGCGGTTCAAGGGTCAATAC | TACGGAATCCATTTGGGAGCAT |
| Immunoprecipitated genomic DNA fragments for ChIP assay | ACTTCGACGTCATGTTTTA | GAGGTGTTTCCCTTTATCA |
| *BmKRP*-sgRNA | TAATACGACTCACTATAGGAAAATGGGTTCAAAGATACCGTTTTAGAGCTAGAAATAGCAAGTTAAAATAAGGCTAGTCCGTTATCAACTTGAAAAAGTGGCACCGAGTCGGTGCT | AAAAGCACCGACTCGGTGCCACTTTTTCAAGTTGATAACGGACTAGCCTTATTTTAACTTGCTATTTCTAGCTCTAAAA |
| *BmKRP* site | GTACTGTGTAGAGTGCATGA | AGGTATTGGCGTTTTAGTGT |

**Figures**


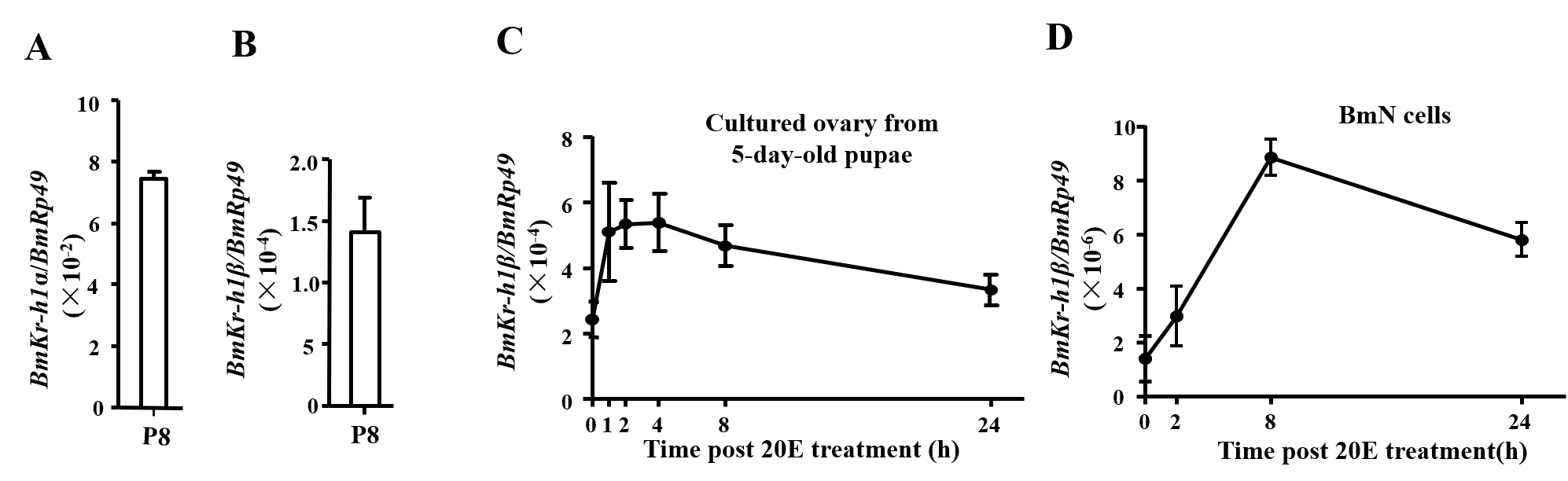


**Additional file, Figure S1. qRT-PCR analysis of the expression and induced expression of *BmKr-h1β* by 20E in cultured ovaries and BmN cells.** *(A)* mRNA expression of *BmKr-h1α* and *BmKr-h1β* *(B)* in the ovaries from 8-day-old pupae. *(C)* Temporal changes of the *BmKr-h1β* expression post 1 µM 20E treatment in the cultured ovaries from 5-day-old pupae and BmN cells *(D)*. *Rp49* amplified from the same RNA samples was used as the internal control.


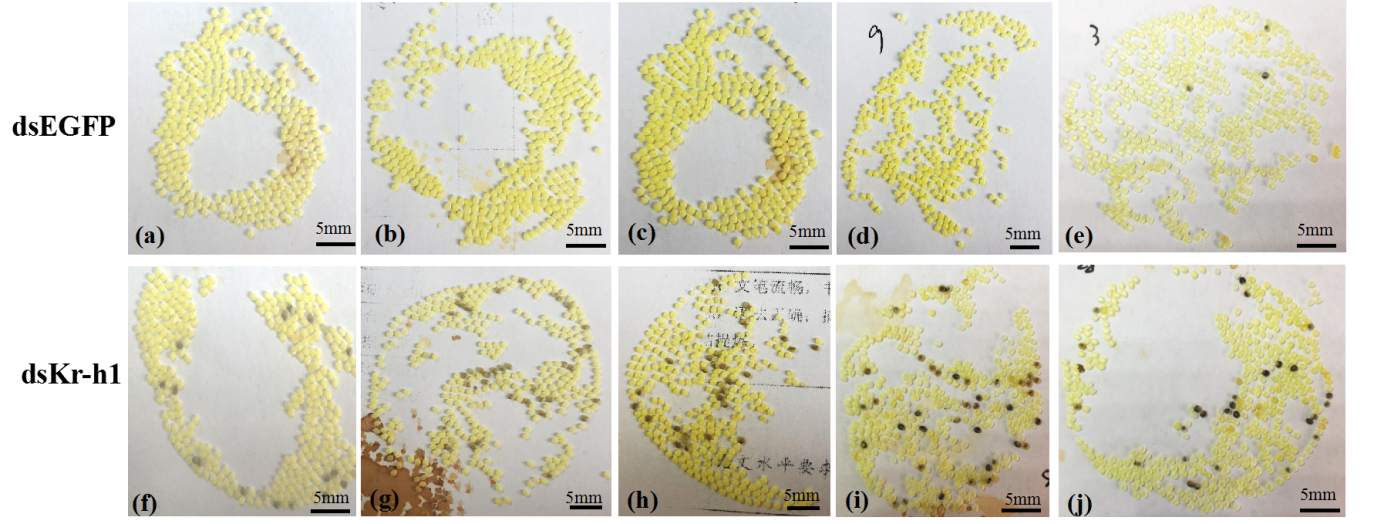


**Additional file, Figure S2. Development of eggs oviposited from the dsRNA-treated adults.** (a)-(e) and (f) to (j) represented day 0 to day 4 of the eggs, respectively.

**
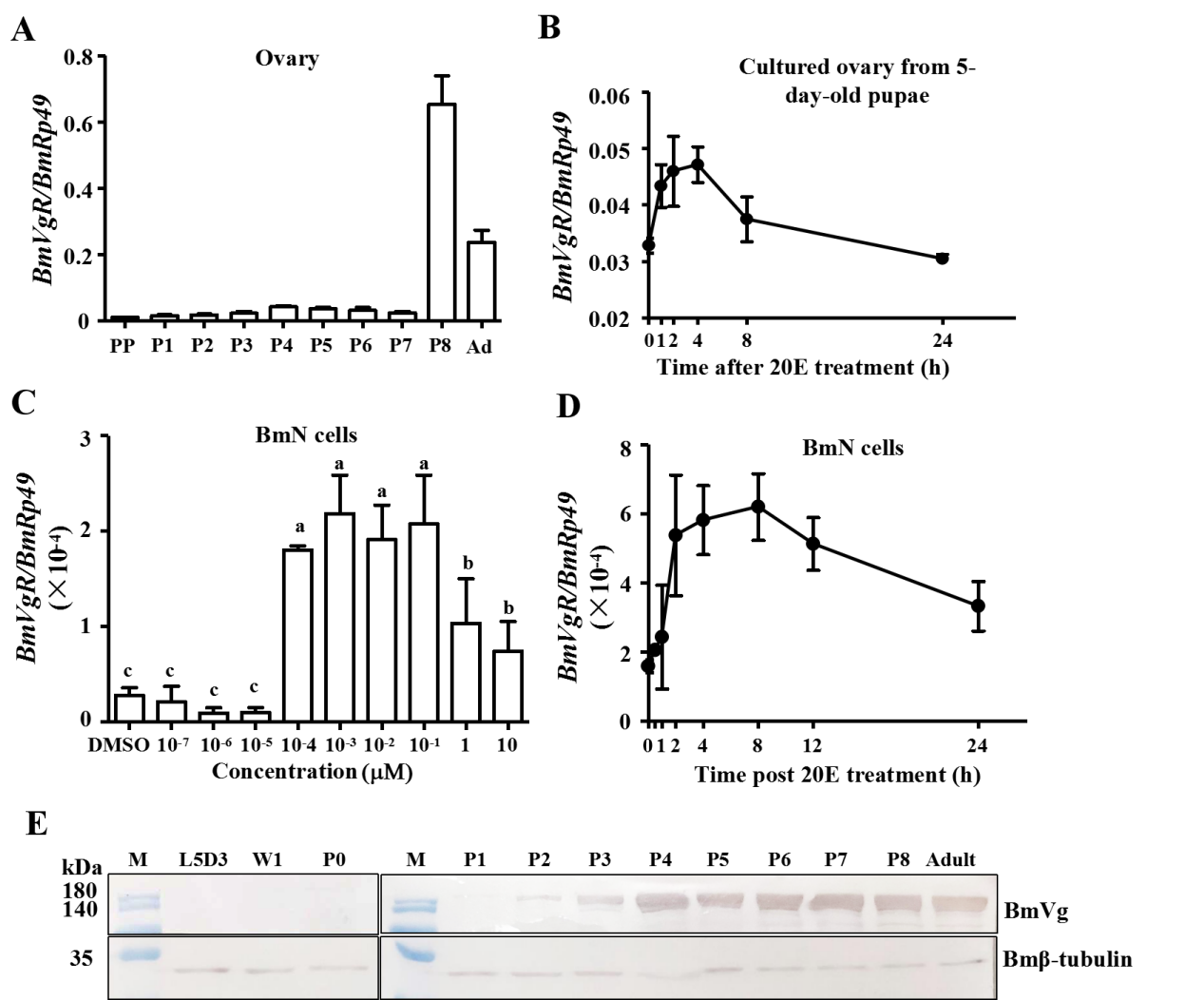
**

**Additional file, Figure S3. Developmental and 20E-induced expression patterns of BmVg and *BmVgR* in the ovaries and BmN cells.** *(A)* Developmental expression proﬁles of *BmVgR* in the ovaries. *(B)* Temporal changes of *BmVgR* expression in the cultured ovaries from 5-day-old pupae. *(C)* Dose-dependent and temporal changes *(D)* of *BmVgR* expression in BmN cells. Data is mean ± SEM (n=3) and the individual data values are shown in Additional file 2. *(E)* Western blotting analysis of BmVg expression in the ovaries. Different letters above the columns indicated significance in the difference of luminescence at *p*<0.05 by ANOVA analysis. M: Prestained Protein Ladder Marker. nD: Day of development; nL: stage of larval instar; W: wandering stage; P: pupal stage.


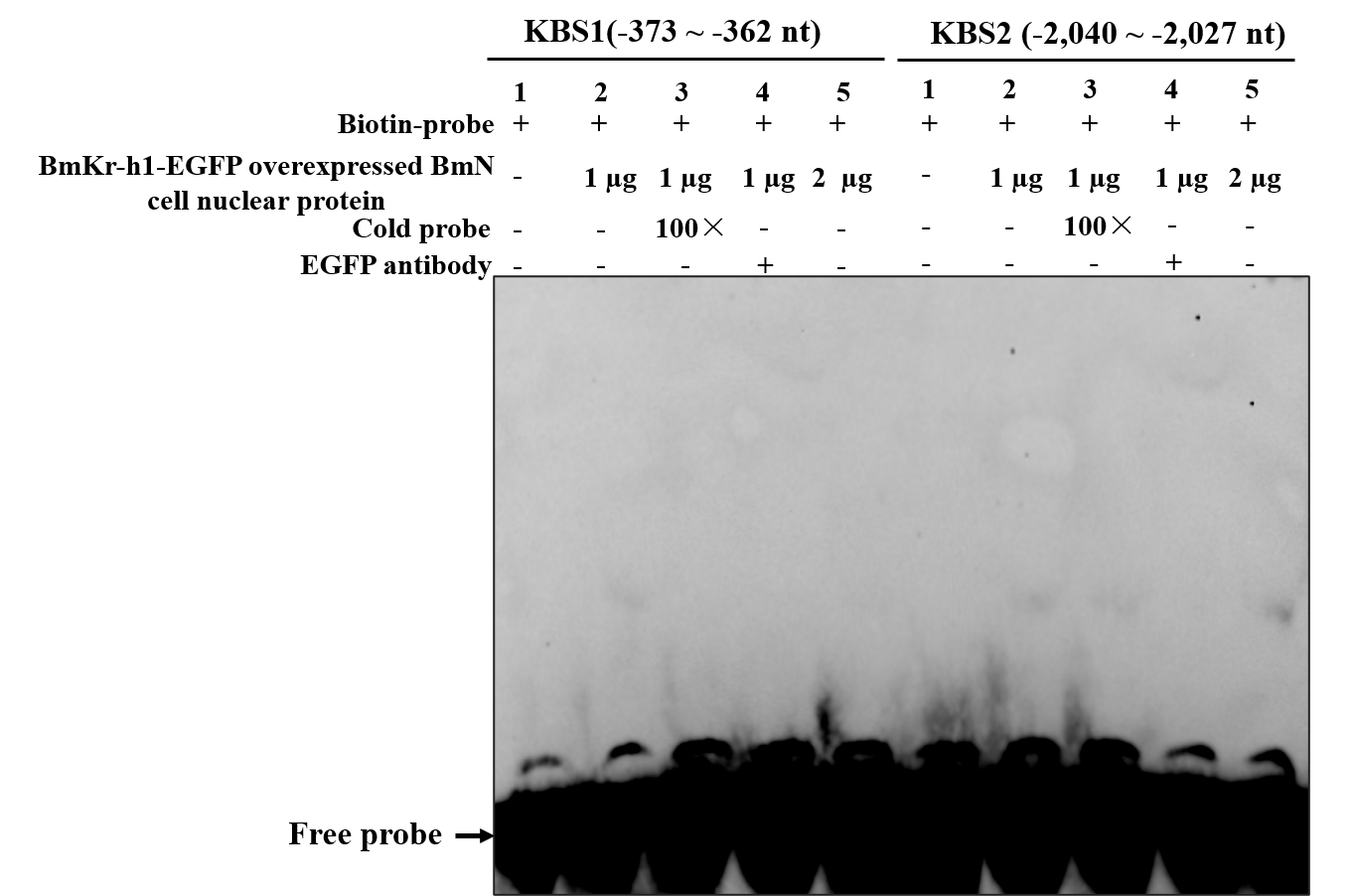


**Additional file, Figure S4. EMSA of the binding of the nuclear protein isolated from BmN cells overexpressing BmKr-h1-EGFP with the KBS1 and KBS2 in the *BmVgR* promoter.** For supershift assay, Anti-EGFP polyclonal antibody was made in a rabbit and used at a dilution of 1:10.

**
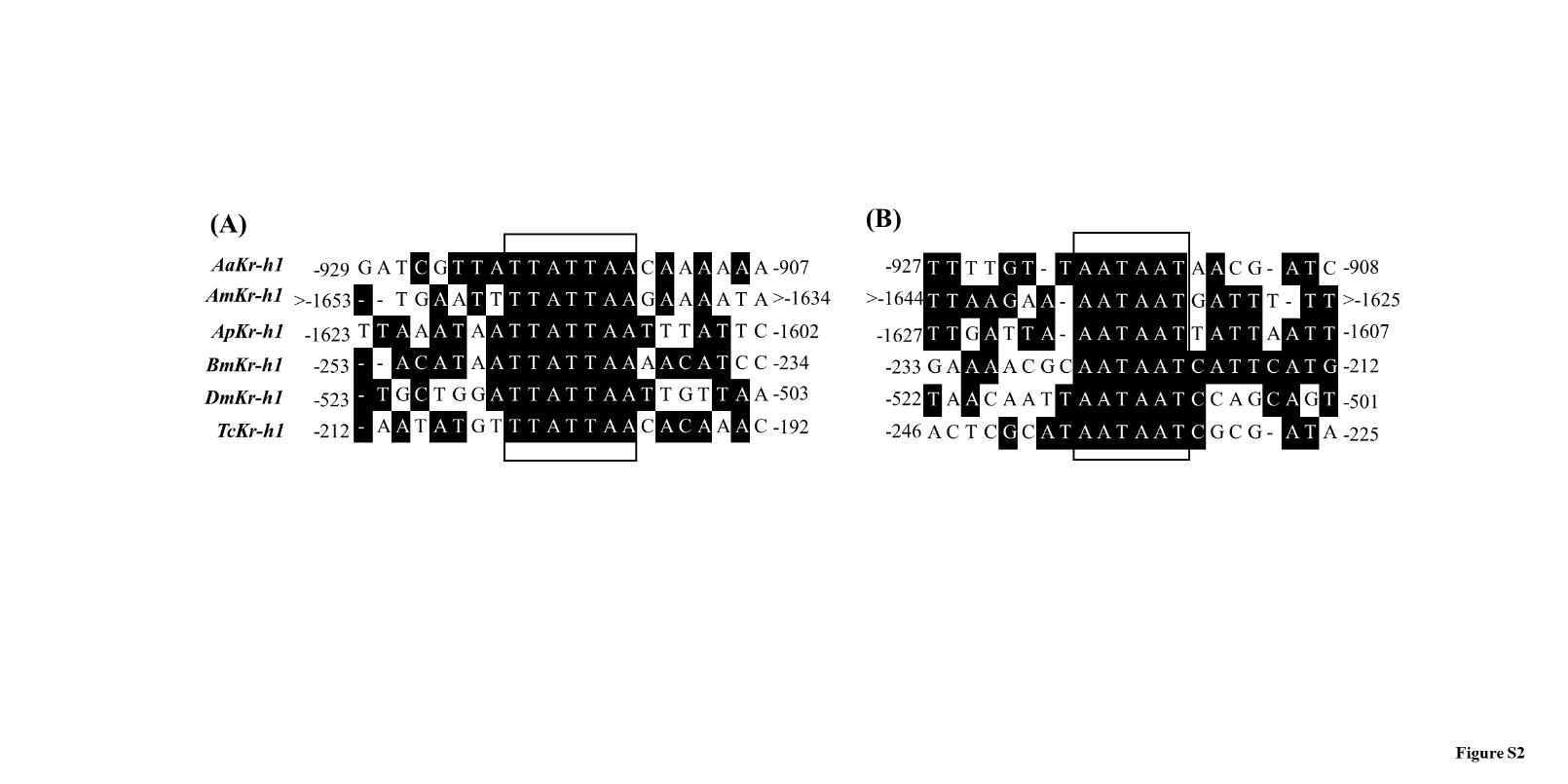
**

**Additional file, Figure S5. Alignment of the putative 20E cis-response elements in the upstream of *Kr-h1* in other insect species.** *Aa*, *Aedes aegypti*; *Am*, *Apis mellifera*; *Ap*, *Acyrthosiphon pisum*; *Bm*, *Bombyx mori*; *Dm*, *Drosophila melanogaster*; *Tc*, *Tribolium castaneum*. The numbers represented the distances from the transcription start site (for *AaKr-h1*, *BmKr-h1*, *TcKr-h1*, *AmKr-h1*, and *DmKr-h1*) or translation start site (for *ApKr-h1*).


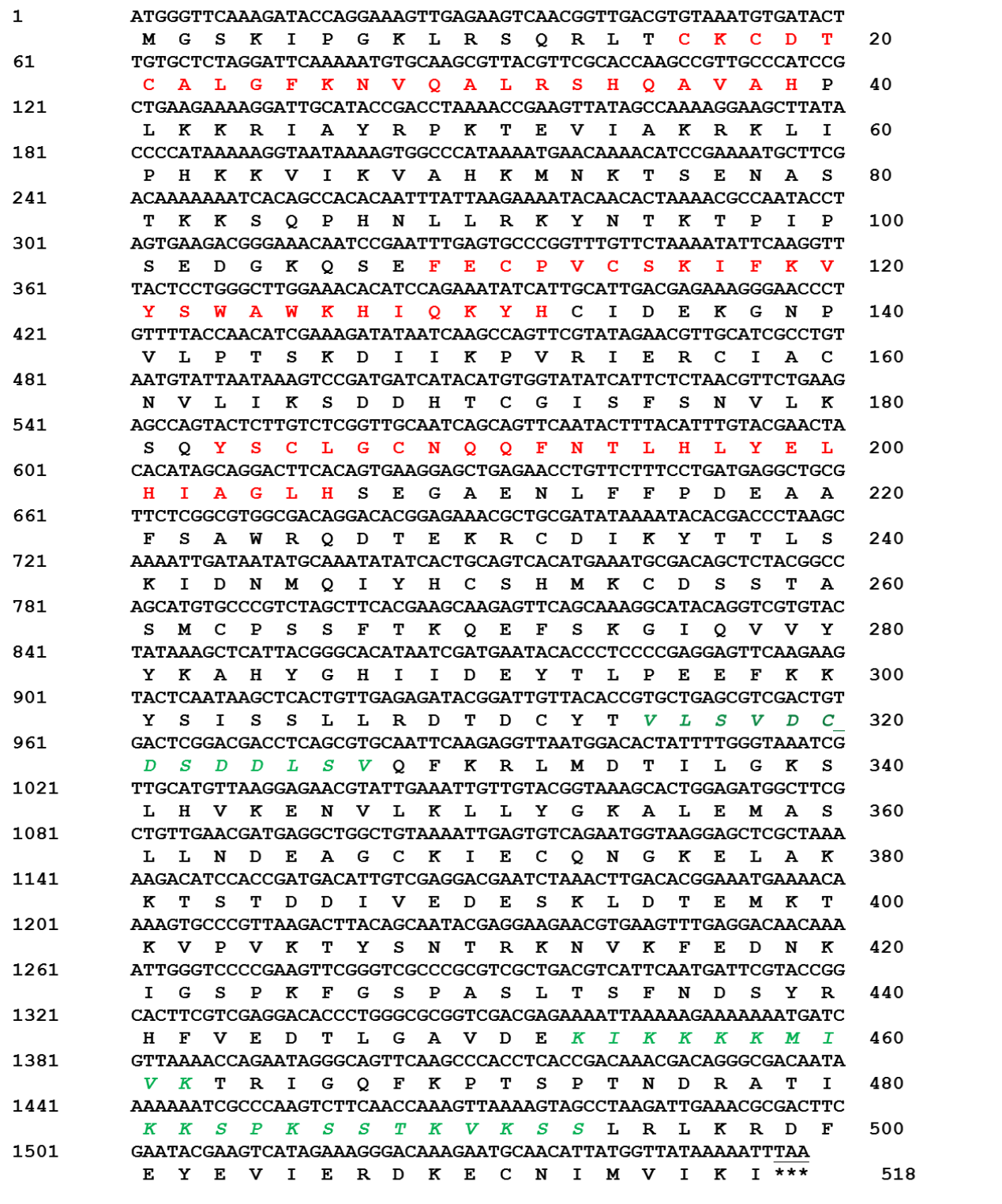


**Additional file, Figure S6. Nucleotide and deduced amino acid sequences of *BmKRP* in *B. mori***. The numbers on the left represented the nucleotide sequence (GenBank accession No.: XM_004922171.3). The numbers on the right represented the amino acid sequence (GenBank accession No.: XP_004922228.1). The stop codon TAA was underlined with stars. The ZnF-C_2_H_2_ domains of *BmKRP* were in red front and the low complexity regions were italicized and in green front.


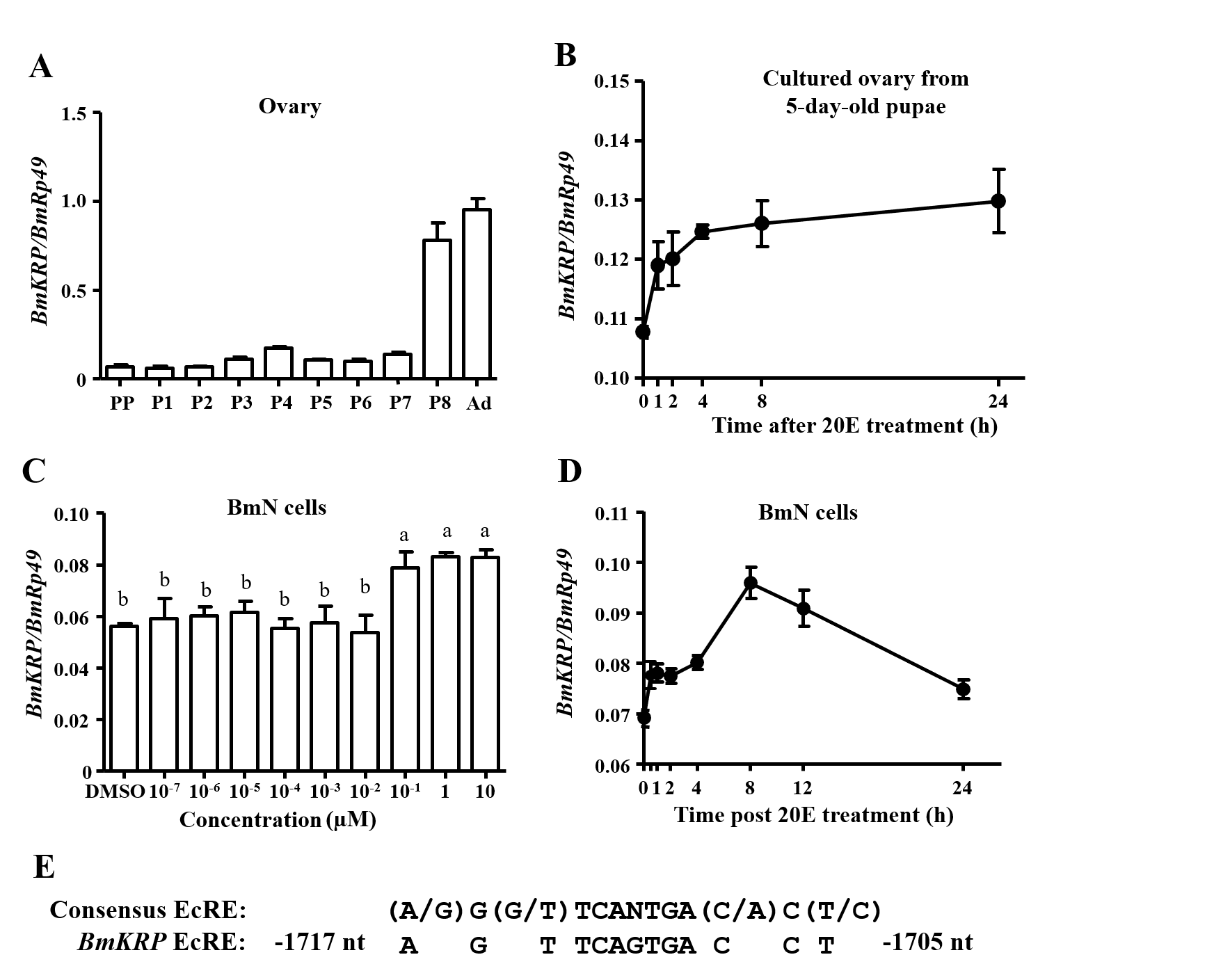


**Additional file, Figure S7. Developmental and 20E-induced expression patterns of *BmKRP* in the ovaries and BmN cells.** *(A)* Developmental expression proﬁles of *BmKRP* in the ovaries. *(B)* Temporal changes of *BmKRP* in the cultured ovaries from 5-day-old pupae after 1 μM 20E treatment. *(C)* Dose-dependent and temporal changes *(D)* of *BmKRP* expression in BmN cell. Data is mean ± SEM (n=3) and the individual data values are shown in Additional file 2. Different letters above the columns indicate significance in the difference of luminescence at p<0.05 by ANOVA analysis. *(E)* Prediction of EcRE in the *BmKRP* promoter based on the conserved sequences of EcRE (33).


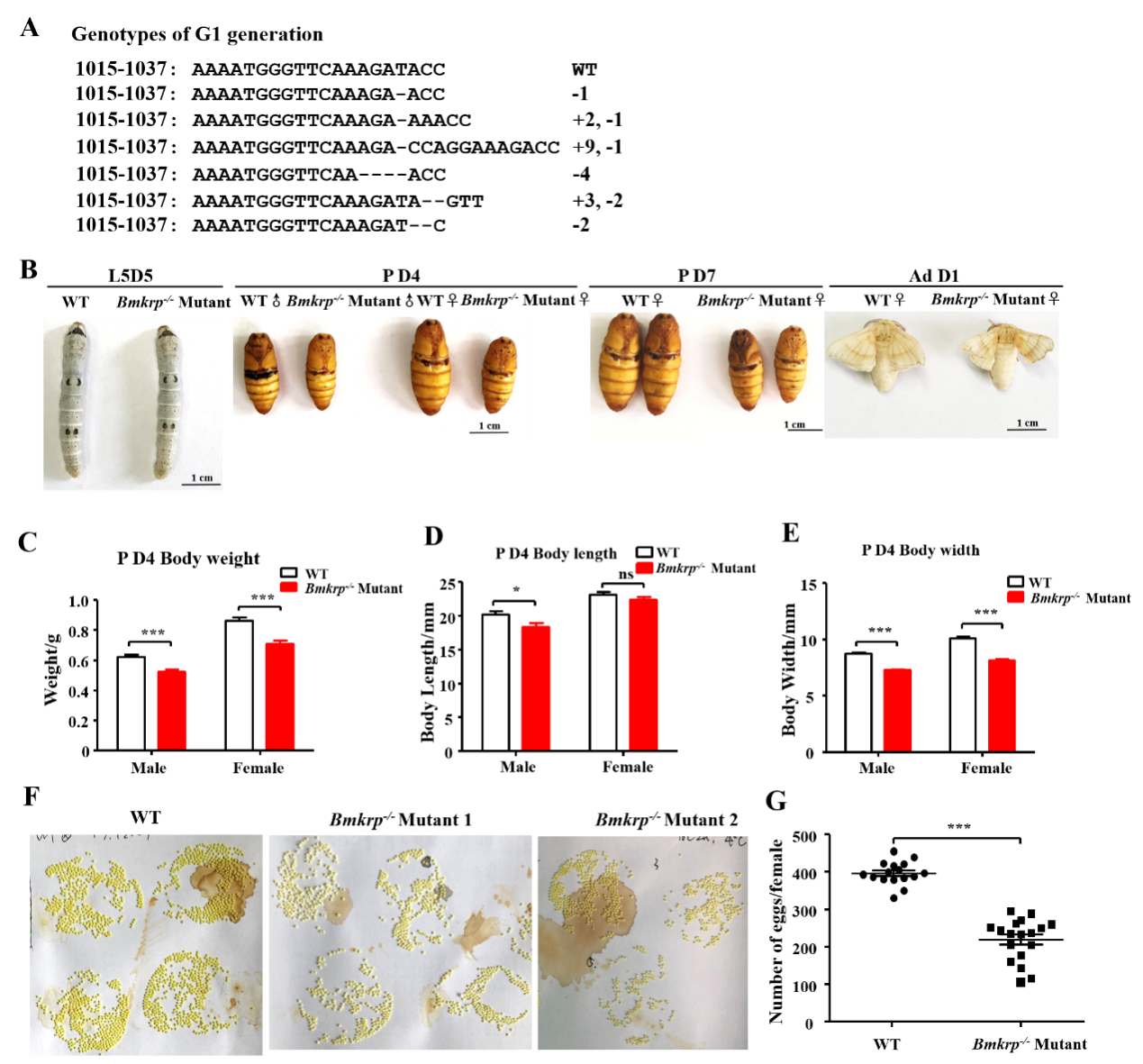


**Additional file, Figure S8. Cas9/sgRNA mediated gene editing of *BmKRP* in the silkworm.** *(A)* The mutant genotypes induced by *BmKRP-*sgRNA in G1 generation. *(B)* Phenotypes of 5^th^ instar larvae at day 5, pupae at day 4 and 7 and adult at day 1. *(C)* Body weight, length *(D)* and width *(E)* of pupae at day 4. *(F)* Eggs laid by wildtype and *Bmkrp^-/-^* mutant female. *(G)* Number of eggs laid by a single female. The significance of the differences between the treatment and control was statistically analyzed at *p* < 0.05 (*), *p* < 0.01 (**) and *p* < 0.001 (***) using *t-*test.
